# MSC‐Derived Exosomal *lnc‐AGT‐3*: A Novel Anti‐Angiogenic Target in Age‐Related Macular Degeneration Through *p53* Signaling Pathway

**DOI:** 10.1111/acel.70377

**Published:** 2026-01-12

**Authors:** Lingjie Kong, Xiaoyan Han, Siyi Qi, Duo Li, Jingyue Zhang, Linyu Zhang, Shujie Zhang, Qin Jiang, Biao Yan, Chen Zhao

**Affiliations:** ^1^ Eye Institute and Department of Ophthalmology, Eye & ENT Hospital Fudan University Shanghai China; ^2^ Key Laboratory of Myopia and Related Eye Diseases, NHC; Key Laboratory of Myopia and Related Eye Diseases Chinese Academy of Medical Sciences Shanghai China; ^3^ Shanghai Key Laboratory of Visual Impairment and Restoration Shanghai China; ^4^ The Affiliated Eye Hospital Nanjing Medical University Nanjing China; ^5^ Department of Ophthalmology, Shanghai General Hospital Shanghai Jiao Tong University School of Medicine Shanghai China; ^6^ Xiamen Eye Center of Xiamen University Xiamen China

**Keywords:** angiogenesis, hnRNP K, *lnc‐AGT‐3*, MSC‐exosomes, neovascular AMD, *p53* signaling pathway

## Abstract

Neovascular age‐related macular degeneration (nAMD) is a major cause of irreversible vision impairment in elderly populations, characterized by pathological angiogenesis beneath the macula. Although anti‐VEGF therapies have demonstrated clinical effectiveness, significant challenges including drug resistance and the need for frequent intravitreal injections persist. As natural nanovesicles, exosomes derived from mesenchymal stem cell (MSC) can mediate intercellular communication, making them an attractive alternative for modulating cellular processes. This study explored the anti‐angiogenic effects of MSC‐derived exosomes in nAMD, with particular emphasis on the role of a specific exosomal lncRNA *lnc‐AGT‐3*. Our results showed that *lnc‐AGT‐3* expression was reduced in both nAMD patients and choroidal neovascularization (CNV) models, and its overexpression effectively inhibited pathological angiogenesis in vitro *and* in vivo*.* Mechanistically, *lnc‐AGT‐3* enhanced the p53 signaling pathway by blocking the ubiquitination and degradation of p53 and ultimately inhibited neovascularization, a process potentially linked to its direct interaction with heterogeneous nuclear ribonucleoprotein K (hnRNP K). Our findings position MSC‐derived exosomes enriched with *lnc‐AGT‐3* as an innovative therapeutic paradigm for nAMD, acting through *p53* pathway modulation to potentially overcome current treatment limitations.

## Graphical abstract

MSC‐derived exosomes deliver lnc‐AGT‐3 to vascular endothelial cells, where it binds hnRNP K in the nucleus. This interaction inhibits p53 ubiquitination, enhancing p53 stabilization and transcriptional activation of anti‐angiogenic targets TSP1. Consequently, p53‐mediated signaling is potentiated, effectively suppressing choroidal neovascularization in AMD.

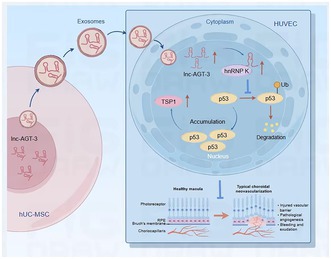

## 1 Introduction

Age‐related macular degeneration (AMD), a progressive retinal disorder, represents the primary cause of permanent vision impairment in adults aged 60 and older globally (Fleckenstein et al. 2021). Current epidemiological projections indicate that approximately 288 million individuals will be affected by 2040, with demographic shifts toward an aging population driving this increasing prevalence (Wong et al. 2014). Advanced AMD progresses through two distinct pathways: non‐neovascular (dry) and neovascular (wet) AMD. The latter, marked by pathological choroidal neovascularization (CNV), is responsible for 90% of advanced vision impairment cases, driven by VEGF‐mediated uncontrolled angiogenesis (Vemulakonda et al. 2025; Yeo et al. 2019). While anti‐VEGF agents (e.g., ranibizumab, aflibercept) demonstrate clinical efficacy, significant limitations persist such as treatment resistance and frequent injections, highlighting the need for alternative therapies (Song et al. 2022; Sunaga et al. 2024).

Mesenchymal stem cells (MSCs) are multipotent stromal cells of mesodermal origin that demonstrate therapeutic potential for age‐related disorders, owing to their self‐renewal capacity, multipotent differentiation potential, and paracrine activity (Fu et al. 2019). MSCs exert their therapeutic effects primarily via their secretome, particularly exosomes, which are nanoscale extracellular vesicles (30–200 nm) carrying bioactive cargo (proteins, lipids, RNAs) that mediate intercellular communication (Ding et al. 2023). Exosomes derived from MSCs (MSC‐exosomes) exhibit broad therapeutic potential across various disease models, spanning cardiovascular, neurological, and ocular pathologies, through their capacity to regulate cellular functions and promote tissue regeneration (Chen et al. 2022; Wu et al. 2023).

Long non‐coding RNAs (lncRNAs), a class of regulatory RNAs exceeding 200 nucleotides in length, have recently gained attention as critical regulators of gene expression at both transcriptional and post‐transcriptional levels (Gonzales et al. 2024). These versatile molecules orchestrate essential cellular functions ranging from proliferation and differentiation to programmed cell death (Ransohoff et al. 2018). Emerging evidence suggests that lncRNAs are specifically sorted into exosomes where they serve as molecular messengers, actively participating in disease progression through cell–cell communication in cancer, metabolic dysfunction, and neurodegeneration (Dai et al. 2025; Liu et al. 2025; Shi et al. 2025). This newly identified signaling paradigm provides transformative insights into disease pathogenesis while offering innovative therapeutic opportunities.

This study tested the hypothesis that MSC‐exosomes regulate pathological neovascularization in nAMD by modulating specific lncRNAs and their downstream molecular targets in vascular endothelial cells. We identified *lnc‐AGT‐3* as a highly enriched lncRNA in MSC‐exosomes that can be efficiently internalized by recipient endothelial cells. Mechanistically, *lnc‐AGT‐3* may activate *p53* signaling via direct binding to heterogeneous nuclear ribonucleoprotein K (hnRNP K), resulting in potent inhibition of pathological angiogenesis both in vitro and in vivo. Furthermore, we deciphered the precise molecular mechanisms by which *lnc‐AGT‐3* regulates p53 expression. To sum up, our findings establish *lnc‐AGT‐3* as a key functional cargo of MSC‐exos and provide a novel anti‐angiogenesis target with potential clinical application for treating nAMD.

## 2 Materials and Methods

### 2.1 Ethics Approval

The research was conducted in accordance with the ethical principles outlined in the Declaration of Helsinki and obtained approval from the Animal Care and Use Committee of the authors' institution (License No. IACUC‐2412066). C57BL/6J and neoretinal vascularization 2 (NRV2) mice (*Crb1* ^*rd8*^ *Jak3* ^*m1J*^/Boc) were sourced from Nanjing Medical University (China) and Jackson Laboratory (USA), respectively. All animals were maintained in a pathogen‐free facility with controlled light/dark cycles (12 h each) at 25°C ± 1°C.

### 2.2 Clinical Sample Collection

This study was approved by the Ethical Committee of the Affiliated Eye Hospital of Nanjing Medical University (2023016). AH samples were collected (2023–2024) at Nanjing Medical University Eye Hospital under Declaration of Helsinki guidelines. nAMD samples were obtained pre–anti‐VEGF injection, while controls (age‐related cataract, ARC) were collected during cataract surgery. Exclusion criteria included: (1) ocular surgery, trauma, inflammation, tumor, or secondary cataract history; (2) prior anti‐VEGF therapy in the study eye; (3) systemic conditions such as active infections (HBV, HCV, syphilis, HIV), psychiatric/hereditary disorders, significant cardiocerebrovascular/hepatic/renal disease, endocrine disorders, or malignancy. The information of ARC patients and nAMD patients involved in the study was shown in Table S1.

### 2.3 Cell Maintenance and Experimental Manipulation

Human umbilical vein endothelial cells (HUVECs) and human umbilical cord‐derived mesenchymal stem cells (hUC‐MSCs) were obtained from ATCC (Manassas, VA, USA) and Nuwacell Biotechnology Co. Ltd. (Anhui, China), respectively. HUVECs were cultured in endothelial cell medium (ECM, ScienCell) supplemented with 10% FBS and 1% penicillin–streptomycin at 37°C with 5% CO_2_. MSCs were cultured in ncMission FBS‐free basal medium (Nuwacell, RP02010) at 37°C with 5% CO_2_. For cell treatment, CHX (100 μg/mL, MCE, HY‐12320) and MG132 (20 μM, MCE, HY‐13259) were incubated with HUVECs for the specified durations as required.

### 2.4 Transfection of Lentivirus, siRNAs and Plasmids

The Ensembl Transcript ID of the human *lnc‐AGT‐3* used in this study is ENST00000444423.2, with genomic coordinates Chr1: 230,612,009–230,612,412 (GRCh38/hg38) and corresponding gene symbol RPS24. A lentiviral containing the full‐length *lnc‐AGT‐3* sequence (404 bp) for overexpression and a matched negative control were provided by OBiO Technology (Shanghai, China). In parallel, knockdown experiments employed a combination of three siRNAs and three ASOs targeting *lnc‐AGT‐3*, along with the respective controls, supplied by Ribobio Corporation (Guangzhou, China). Besides, siRNAs against p53 and hnRNP K and their controls were custom‐synthesized by GeneAdv Corporation (Suzhou, China). Truncated hnRNP K plasmids with 3X Flag c‐tag were also synthesized by OBiO Technology, with the empty pcDNA3.1 vector serving as a control. Moreover, transfections were conducted using Lipofectamine 3000 (for plasmids) and Lipofectamine RNAiMAX (for siRNAs/ASOs) (Invitrogen, USA), following the manufacturer's protocol. Final concentrations were standardized to 80 nM for *lnc‐AGT‐3* siRNA, 50 nM for *p53*, *hnRNP K*, and *TSP1* siRNAs, and 2.5 μg/mL for plasmids. Detailed oligonucleotide sequences were documented in Tables S2 and S3.

### 2.5 Exosome Isolation and Characterization

Prior to the commencement of the experiment, MSCs were cultured until they reached the 3rd passage. Subsequently, exosomes were extracted from MSC culture medium using an exosome isolation kit (EZB, USA, EZB‐exo201‐S). The collected cell culture medium was subjected to centrifugation at 3000 × *g* for 10 min at 4°C to eliminate cells and debris. Subsequently, the clarified supernatant was then mixed with exosome precipitation reagent at a 4:1 ratio (medium: reagent) and incubated overnight at 4°C. Following incubation, samples were centrifuged at 10,000 × *g* (4°C, 30 min) to pellet the exosomes. After removing the supernatant, the exosome pellets were gently resuspended in PBS for downstream applications.

The identification of exosomes includes the size, shape, diameter, quantity, and the specific markers. Firstly, morphological assessment was performed using transmission electron microscopy (TEM) (Hitachi, Japan, HT‐7700). Then, quantitative analysis of particle size and concentration was conducted by nanoparticle tracking analysis (NTA) (PARTICLE METRIX, Germany, ZetaVIEW). Additionally, western blotting detected characteristic exosomal markers including CD9 (ab263019), Hsp70 (ab181606), and TSG101 (ab125011) (all antibodies from Abcam, diluted 1:1000), while Calnexin (Abcam, ab133615) served as a negative control to verify exosome purity.

For protein characterization, exosomal lysates were separated via SDS‐PAGE (Yamay, China) and subsequently electrotransferred onto PVDF membranes (Millipore, Germany). Following immunodetection, target proteins were visualized by enhanced chemiluminescence and quantified through densitometric analysis (ImageJ). Total exosomal protein content was determined by a BCA assay (Beyotime, China), with all measurements normalized to the total protein concentration.

### 2.6 Laser‐Induced CNV in Mice and Intravitreal Delivery

Six‐ to eight‐week‐old C57BL/6J mice were anesthetized by intraperitoneal injection of a mixture of 10% tiletamine, 10% zolazepam, and 2% xylazine at a dosage of 0.1 mL per 10 g of body weight. Subsequently, the pupils were dilated with 0.5% tropicamide. Laser photocoagulation was performed using a 532 nm laser system (VITRA, Quantel medical, France, CS40015) with the following parameters: 130 mW power, 100 ms duration, and a spot size of 50 μm. A sterile glass coverslip coupled with carbomer gel was applied to the cornea to flatten the corneal surface and focus the laser beam onto the retinal pigment epithelium. Each eye was subjected to four separate laser burns, which were evenly distributed around the optic disc and positioned away from blood vessels. Successful Bruch's membrane rupture was verified by the immediate formation of a distinct heat bubble at the site of injury. For intravitreal injection, 0.5% tetracaine hydrochloride was applied to the corneas for surface anesthesia, followed by 0.5% tropicamide for pupil dilation. A Hamilton microinjector (Reno, NV, USA) was utilized to carry out the intravitreal injection at a location 1 mm posterior to the limbus. Finally, levofloxacin eye ointment was applied to prevent bacterial infection. Following surgical procedures, mice were maintained on a temperature‐regulated warming platform (37°C) throughout the recovery period to prevent anesthesia‐induced hypothermia.

### 2.7 AAV‐Mediated Ocular Gene Delivery in Murine Models

We developed an AAV2/9‐based gene delivery system for ocular‐specific overexpression of *lnc‐AGT‐3* (AAV‐*AGT*), with two distinct vector designs tailored for different experimental requirements. In C57BL/6 mice (18–22 g), intravitreal injections of 2 μL virus solution (2 × 10^9^ GC/eye) were administered to achieve sustained transgene expression. For accelerated expression in NRV2 mice, we utilized a self‐complementary AAV vector (scAAV‐*AGT*) delivered at postnatal Day 18, enabling robust *lnc‐AGT‐3* overexpression within 4 days post‐injection. Self‐complementary AAV2/8 vectors carrying shRNA against *TSP1* were constructed for intravitreal delivery to achieve targeted gene silencing in mouse retinal tissue. All viral vectors, including matched control constructs (AAV‐MCS, scAAV‐MCS, and Vector), were custom‐designed and produced by OBiO Technology Corp. Ltd. located in Shanghai, China.

### 2.8 Fundus Photography and Fluorescein Angiography (FFA)

Seven days post‐injection, mice underwent comprehensive retinal evaluation using the OPTO‐RIS imaging system (Optoprobe, UK). Following anesthesia and pupillary dilation, animals were positioned to ensure proper corneal contact with the imaging objective. Vascular integrity was assessed via fluorescein angiography, with 100 μL of 2.5% sodium fluorescein (Alcon Laboratories, USA) administered intraperitoneally. Digital images acquired 3–5 min post‐injection enabled quantitative analysis of pathological neovascularization through measurement of fluorescein leakage areas.

### 2.9 Optical Coherence Tomography (OCT)

The ISOCT system (Optoprobe, UK) was employed for high‐resolution cross‐sectional imaging of retinal architecture in treated mice. Centered on the optic disk, vertical scans enabled detailed segmentation of three distinct retinal strata: (1) the inner retinal complex (RNFL, GCL, and IPL), (2) the middle retinal compartment (INL, OPL, ONL, and OLM), and (3) the photoreceptor‐RPE complex (IS/OS segments and retinal pigment epithelium). This layered quantification protocol provided precise morphological assessment of treatment effects across all major retinal substructures.

### 2.10 Electroretinography (ERG)

The retinal function of mice was evaluated using electroretinography (ERG). ERG testing was carried out following an overnight dark adaptation period. Animals were positioned on a thermoregulated platform throughout the procedure, which was conducted under dim red‐light illumination to preserve dark adaptation. Using a three‐electrode configuration (corneal active, cheek reference, tail ground), we obtained simultaneous bilateral recordings across a clinically relevant intensity range (0.01–10 cd.s/m2). The Diagnosys 6.63 platform automatically quantified photoreceptor (a‐wave) and bipolar cell (b‐wave) responses, enabling comprehensive evaluation of retinal signal transduction pathways.

### 2.11 Ex Vivo Choroidal Sprouting Assay

Eyes from euthanized C57/BL6 mice were rapidly enucleated and maintained in chilled DMEM for processing. Following removal of anterior segment structures, the posterior pole was microdissected to isolate peripheral RPE‐choroid‐sclera complexes, which were sectioned into 1 mm^2^ explants. These tissue fragments were embedded in growth factor‐reduced Matrigel (40 μL/well; BD Biosciences, USA, 354230) within 24‐well plates and cultured in DMEM supplemented with 10% FBS and antibiotics under standard conditions (37°C, 5% CO_2_). Angiogenic sprouting was monitored from Days 3 to 5 post‐embedding using phase‐contrast microscopy (Olympus IX70, Tokyo, Japan), with quantitative analysis of neovascular outgrowth performed via ImageJ.

### 2.12 Biotin RNA Pull‐Down and Mass Spectrometry Analysis

RNA pull‐down assays were performed to identify *lnc‐AGT‐3* binding partners (Yao et al. 2022). Briefly, cell lysates prepared in RNase‐free lysis buffer were incubated with biotinylated *lnc‐AGT‐3* or antisense RNA (RiboBio, China) overnight at 4°C. RNA‐protein complexes were captured using streptavidin magnetic beads (Smart‐Lifesciences, SM017001) with rotation (2 h, RT), followed by extensive washing to remove non‐specific interactions. Bound proteins were subsequently subjected to western blotting or mass spectrum analysis. Different bands pulled by *lnc‐AGT‐3* and antisense RNA were detected by Silver Stain Kit (Beyotime, China, P0017S) and cut for the following mass spectrometry analysis. The *lnc‐AGT‐3* probe sequences were provided in Table S4.

### 2.13 RNA Immunoprecipitation (RIP) Analysis

RNA‐protein interactions were investigated using the Magna RIP Kit (Merck, Germany, 17–700). Cell lysates were incubated with hnRNP K antibody‐conjugated magnetic beads (Proteintech, 11,426–1‐AP, 5 μg) or control IgG (Merck, PP64B) at 4°C overnight. The immunoprecipitated RNAs were then purified using Trizol and ethanol precipitation and subjected to qPCR analysis of *lnc‐AGT‐3* expression.

### 2.14 Agilent Human ceRNA Microarray

Transcriptomic profiling of HUVECs under oxidative stress (H_2_O_2_ stimulation) and MSC‐exosome treatment was performed using Agilent Human ceRNA Microarray 2019 (4 × 180 K, Design ID: 086188) by OE Biotech Co. Ltd. (Shanghai, China). Following RNA quality verification (NanoDrop ND‐2000; Agilent, Bioanalyzer 2100), samples were processed through cDNA synthesis, Cy3‐labeled cRNA preparation, and array hybridization according to the manufacturer's protocols. Array scanning (Agilent, G2505C) and initial data processing (Feature Extraction, 10.7.1.1) were followed by quantile normalization and probe filtering (≥ 75% detection rate). Differential expression thresholds were set at |FC| ≥ 2.0 with *p* ≤ 0.05 (Student's *t*‐test), with subsequent functional annotation through GO and KEGG pathway analyses. Hierarchical clustering visualized distinct expression patterns across experimental conditions. The raw microarray data have been deposited in NCBI's Gene Expression Omnibus (GEO) under accession number GSE299892.

### 2.15 mRNA Sequencing

RNA sequencing of *lnc‐AGT‐3*‐silenced HUVECs (24 h post‐transfection, *n* = 3) was conducted on the Illumina NovaSeq 6000 platform (150 bp paired‐end) at OE Biotech Co. Ltd. (Shanghai, China). Approximately 54 million raw reads per sample underwent quality filtering (fastp) and adapter trimming, yielding ~49 million clean reads. Processed reads were aligned to the reference genome (HISAT2) and quantified (HTSeq‐count/FPKM). Quality control included PCA evaluation of biological replicates using R (v3.2.0). Differential expression analysis (DESeq2) identified significant transcripts (|FC| ≥ 2, *p* < 0.05), visualized through hierarchical clustering and radar plots (ggradar). Functional annotation incorporated GO, KEGG, Reactome and WikiPathways enrichment analyses (hypergeometric test), with results displayed via multiple visualization methods (column/chord/bubble plots). Complementary GSEA examined predefined gene set enrichment across expression rankings. The datasets supporting this study are publicly available in the NCBI repository (Accession: PRJNA1267020).

### 2.16 Statistical Analysis

Quantitative data are presented as mean ± Standard Error of the Mean (SEM) from ≥ 3 biological replicates. Comparative analyses were performed using appropriate statistical tests: Student's *t*‐test or Mann–Whitney *U* test for two‐group comparisons, and one‐way ANOVA with post hoc testing for multi‐group analyses. For paired observations in NRV2 mice (e.g., depigmentation/leakage measurements), paired *t*‐tests or Wilcoxon signed‐rank tests were applied. All analyses were conducted at *p* < 0.05 significance threshold using a suite of analytical tools (GraphPad Prism 8.0 for graphing, SPSS 25 for advanced statistics, Adobe Illustrator 2020 for figure refinement).

## 3 Results

### 3.1 Characterization of MSC‐Derived Exosomes

**FIGURE 1:**
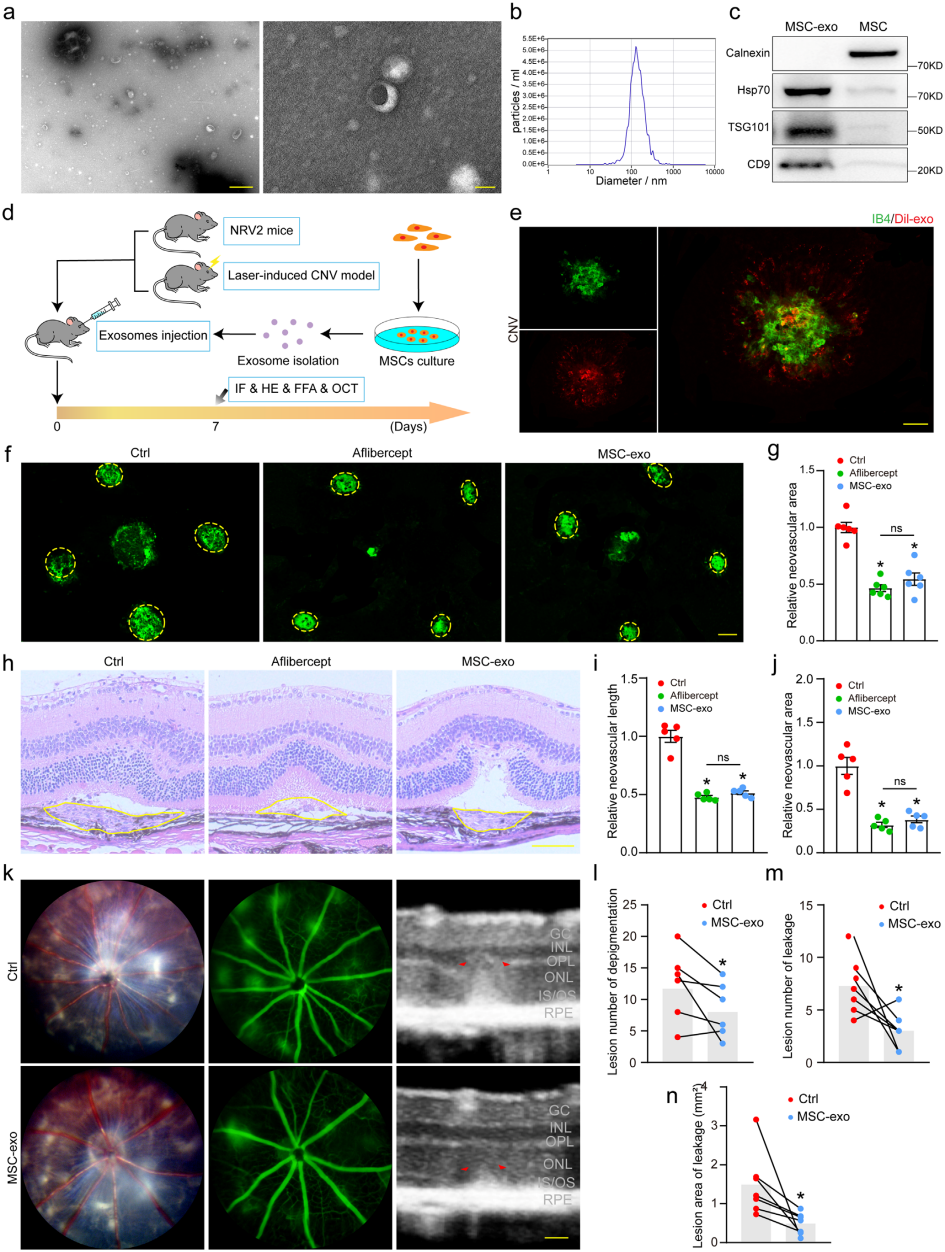
MSC‐exosomes play an anti‐angiogenic role in choroidal neovascularization. (a) TEM images of hUC‐MSC‐derived exosomes (Scale bar, left‐500 μm, right‐50 nm). (b) NTA profile showing exosome size distribution. (c) Western blot analysis of exosomal markers (HSP70, TSG101, CD9) and negative control (Calnexin). (d) Schematic of experimental design for evaluating MSC‐exo therapy in laser‐induced CNV and NRV2 (*Crb1* ^*rd8*^ *Jak3* ^*m1*J^/Boc) mouse models. (e) Three days post‐laser photocoagulation and intravitreal injection of Dil‐labeled exosomes (red), RPE/choroid complexes were isolated and flat‐mounted for IB4 staining (green). (f, g) IB4‐labeled flatmounts of RPE/choroid complexes 7 days post‐laser. Yellow circles denote CNV lesions. Treatments: MSC‐exos (50 μg), aflibercept (40 mg/mL), or PBS control (2 μL intravitreal injections). Four spots per eye were averaged (*n* = 6, Scale bar, 200 μm). *p < 0.05 versus Ctrl group; “ns” represents no statistical significance between the marked group (One‐way ANOVA with Bonferroni's test). (h–j) HE‐stained retinal sections showing reduced CNV length/area after exosome treatment (*n* = 6, Scale bar, 200 μm). *p < 0.05 versus Ctrl group; “ns” represents no statistical significance between the marked group (One‐way ANOVA with Bonferroni's test). (k–n) Fundus photographs, fluorescein angiography and OCT of NRV2 mice after intravitreal injections of PBS (2 μL, left eye) and MSC‐exos (2 μL, 50 μg, right eye) for 7 days (*n* = 7, Scale bar, 50 μm). *p < 0.05 versus Ctrl group (Paired *t*‐test/Wilcoxon test).

To confirm the identity and purity of isolated extracellular vesicles, MSC‐derived exosomes were subjected to comprehensive characterization. Transmission electron microscopy (TEM) revealed the typical cup‐shaped morphology of exosomes, with diameters averaging ~100 nm (Figure 1a). Nanoparticle tracking analysis (NTA) demonstrated a size distribution of 84–223 nm, with a predominant peak at 149 nm, consistent with exosomal dimensions (Figure 1b). Western blotting confirmed the enrichment of exosome‐specific markers (CD9, Hsp70, TSG101) in purified vesicles, while the endoplasmic reticulum protein Calnexin was absent—further validating the exclusion of cellular contaminants (Figure 1c).

### 3.2 MSC‐Exosomes Modulate Angiogenesis In Vivo

To evaluate the therapeutic potential of MSC‐derived exosomes (MSC‐Exos) in nAMD, we administered intravitreal injections of purified MSC‐Exos (Figure 1d). It allowed for direct delivery of exosomes to the retinal microenvironment, enabling investigation of their effects on pathological angiogenesis and disease progression in a controlled manner. Firstly, we administered dil‐labeled exosomes into the eyes of mice following laser treatment to examine the uptake of exosomes by ocular tissues. Subsequent fluorescence imaging was conducted 3 days post‐injection, demonstrating the accumulation of exosomes within the lesion and adjacent regions (Figure 1e; Figure S1). After that, we employed a laser‐induced CNV model to assess the impact of MSC‐exos on neovascularization on Day 7 after laser injury. The results unveiled that MSC‐exos reduced the lesion area in laser‐induced CNV mouse model, and showed an analogous effect of anti‐VEGF agent (aflibercept) on CNV inhibition (Figure 1f,g). Similarly, H&E staining revealed that CNV lesion length and area were notably decreased 7 days after laser‐induced damage (Figure 1h–j).

Then the NRV2 model, a genetically driven system characterized by spontaneous retinal neovascularization mimicking advanced human proliferative retinopathy, was also used to validate the role of MSC‐exos in neovascularization. Previous research has shown that widespread depigmentation in the retina starts to be noticeable around postnatal Day 17 (p17), and by p25, these lesions become more clearly defined and increase in size. This depigmented region is linked to the emergence of vascular leakage, which is a key feature of the disease's vascular manifestation (Hasegawa et al. 2014). Intravitreal injections of MSC‐exos were administered at postnatal Day 18 (p18), and subsequent fundus imaging, FFA and OCT were conducted at p25 to provide an accurate assessment of the fundus condition. The findings indicated that in comparison with the control eyes, less depigmented area and leakage were observed in the eyes receiving exosome injection (Figure 1k–n). Meantime, the OCT assessment validated the presence of a highly reflective area, along with irregularities in the outer nuclear layer (ONL) and outer plexiform layer (OPL), in addition to the subretinal and inner segment/outer segment (IS/OS) regions, whereas exosome administration evidently diminished the abnormal area after 1 week (Figure 1k).

Given its critical role in regulating ocular neovascularization, we next investigated whether MSC‐exos had the potential adverse effects on retinal ganglion cells (RGCs) and photoreceptors in the retina. Immunofluorescence staining was conducted to assess the influence of exosomes on retinal neuronal preservation and photoreceptor degeneration by injections of MSC‐exos for 30 days. Compared with PBS group, no significant adverse effects on RGC survival and photoreceptor degeneration were observed in MSC‐exos group as evidenced by the stable fluorescence signals of NeuN‐immunolabeled RGCs and rhodopsin‐expressing photoreceptors (Figure S2a). Consistently, RBPMS staining, a method to detect RGC survival, demonstrated no notable disparities between the two groups (Figure S2b). Furthermore, TUNEL assays confirmed that MSC‐exos did not induce detectable apoptosis in retina (Figure S2c). These findings suggest that the administration of MSC‐exos does not lead to significant detrimental effects on RGCs and photoreceptors histologically. Then, we evaluated its effects on retinal morphology and visual function. Thirty days following the administration of MSC‐exos into the vitreous cavity, retinal thickness in the vicinity of the optic nerve was assessed via OCT. The analysis revealed no statistically significant differences between the MSC‐exos group and PBS group (Figure S3a). Furthermore, an electroretinogram (ERG) analysis was conducted as well to explore the influence of MSC‐exos on the visual functions of mice. The findings indicated that the injection of exosomes did not result in a reduction of the a‐ and b‐wave amplitudes in scotopic ERG, as compared to the control group (Figure S3b–f). Taken together, MSC‐exos can inhibit the abnormal angiogenesis in vivo, but do not damage the retinal morphology and visual function.

### 3.3 MSC‐Exosomes Regulate Angiogenic Effects In Vitro and Are Enriched With *lnc‐AGT‐3*

**FIGURE 2:**
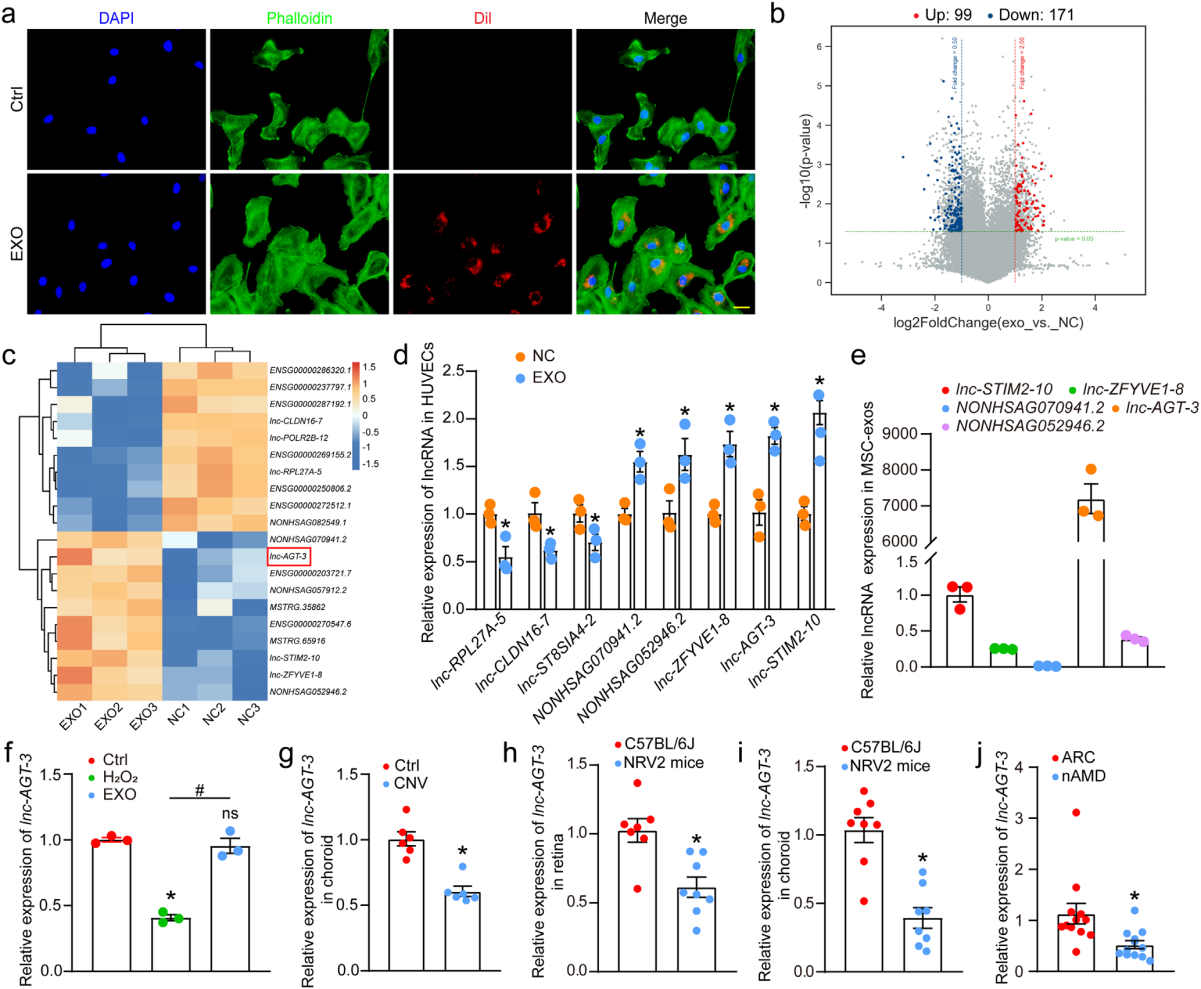
MSC‐exosomes regulate angiogenic effects in vitro and are enriched with *lnc‐AGT‐3*. (a) Cellular uptake of Dil‐labeled MSC‐exosomes (red) in HUVECs, with F‐actin stained by phalloidin (green). Scale bar, 20 μm. (b) Volcano plot of differentially expressed lncRNAs in H_2_O_2_‐stimulated HUVECs NC group and exosome coculture group. (c) Heatmap of the top 10 up−/down‐regulated lncRNAs from microarray. (d) qRT‐PCR validation of microarray data (*n* = 3, *p < 0.05 vs. NC, Student *t* test). (e) lncRNA expression profile in MSC‐exosomes by qRT‐PCR (*n* = 3). (f) HUVECs were exposed to H_2_O_2_ (5 μM) for 24 h, then cocultured with MSC‐exos for another 24 h (Exo) or not (H_2_O_2_). Group Ctrl was left untreated. The levels of *lnc‐AGT‐3* expression were detected by qRT‐PCR assays. (*n* = 3, *p < 0.05 vs. Ctrl, #*p* < 0.05 between the marked group, “ns” represents no statistical significance, ANOVA with Bonferroni). (g) qRT‐PCR assays were conducted to compare *lnc‐AGT‐3* expression in CNV choroids and non‐CNV choroids (*n* = 6, *p < 0.05 vs. Ctrl, Mann–Whitney test). (h, i) Retinal/choroidal *lnc‐AGT‐3* expression in C57BL/6J versus NRV2 mice (*n* = 8, *p < 0.05 vs. C57BL/6J, Student *t* test). (j) *lnc‐AGT‐3* in aqueous humor of nAMD versus ARC patients (*n* = 12, *p < 0.05 vs. ARC, Mann–Whitney test).

Factors capable of exerting anti‐angiogenic effects captured our interest. To explore this further, we selected HUVECs as an in vitro model system to study vascular endothelial cell biology. Firstly, we investigate whether MSC‐exos could be taken in by HUVECs. We utilized dil dye—a fluorescent marker that stains membrane structures and emits red fluorescence under excitation at 549 nm to label the exosomes. After treatment with dil‐stained exosomes for 24 h, microscopic observation revealed clear red fluorescence surrounding the nuclei (Figure 2a). Similarly, CD63 is also used to confirm that exosomes can be internalized by HUVECs (Figure S4a). Then, we employed a low concentration of hydrogen peroxide to treat HUVECs, thereby simulating the pathological conditions characteristic of nAMD. After co‐culturing with MSC‐exos, the hydrogen peroxide‐induced enhancement of cellular proliferation, migration, and tube‐forming capacity was significantly suppressed (Figure S4b–d). To determine the key mediators responsible for the anti‐angiogenic activity of MSC‐exos, we performed transcriptome profiling using the Agilent Human ceRNA Microarray 2019 (4 × 180 K, Design ID: 086188) for HUVECs stimulated by H_2_O_2_ and cocultured with MSC‐exos or not. The differential expression profiling identified 270 dysregulated long non‐coding RNAs (lncRNAs), with 99 showing increased expression and 171 exhibiting decreased levels (Figure 2b). Among these, a heat map was depicted to show the top 10 upregulated and downregulated lncRNAs (Figure 2c). Furthermore, qRT‐PCR was performed to validate the credibility and consistency between microarray and qRT‐PCR results. The results showed that *lnc‐RPL27A‐5*, *lnc‐CLDN16‐7*, and *lnc‐ST8SIA4–2* expression were markedly downregulated, while *NONHSAG070941.2*, *NONHSAG052946.2*, *lnc‐ZFYVE1‐8*, *lnc‐AGT‐3* and *lnc‐STIM2‐10* expression was upregulated in HUVECs treated with MSC‐exos, which correlate well with the microarray profiling results (Figure 2d). To characterize the expression patterns of distinct lncRNAs in MSC‐exos, we conducted qRT‐PCR experiments. The results that only *lnc‐AGT‐3* had abundant expression in MSC‐exos rather than other lncRNAs caught our attention (Figure 2e). In summary, MSC‐exos could regulate angiogenic effects in vitro and are enriched with *lnc‐AGT‐3*.

### 3.4 *lnc‐AGT‐3* Showed Consistent Decreasing in Experimental Neovascularization

Considering the high expression of *lnc‐AGT‐3* in both exosomes and cells, we next explored *lnc‐AGT‐3* expression in pathological model in vitro and in vivo. To mimic the pathological environment of nAMD, H_2_O_2_ was used to cause oxidative damage for HUVECs. qRT‐PCR analysis confirmed significant downregulation of *lnc‐AGT‐3* in HUVECs after H_2_O_2_ stimulation, but experienced a resurgence when treated with MSC‐exos (Figure 2f). Laser‐induced CNV serves as an established experimental system modeling the characteristic vascular abnormalities of nAMD (Lambert et al. 2013). Laser‐induced injury significantly suppressed *lnc‐AGT‐3* levels in RPE/choroid tissues, as confirmed by qRT‐PCR (Figure 2g). To further investigate the expression of *lnc‐AGT‐3* in the ocular tissues of NRV2 mice, qRT‐PCR was performed to detect the expression of *lnc‐AGT‐3* in the retinas and choroids of the mice. The findings revealed a marked decrease in *lnc‐AGT‐3* expression within the retinas and choroids of NRV2 mice compared to that of normal C57BL/6J mice (Figure 2h,i). Finally, to evaluate the clinical significance of *lnc‐AGT‐3* in nAMD, we analyzed 24 aqueous humor (AH) samples from age‐related cataract (ARC, *n* = 12) and nAMD patients (*n* = 12). qRT‐PCR demonstrated significantly reduced *lnc‐AGT‐3* expression in nAMD patients relative to ARC controls (Figure 2j). Collectively, our findings demonstrate consistent downregulation of *lnc‐AGT‐3* across experimental models of neovascularization, implicating its potential role in angiogenic inhibition.

### 3.5 *lnc‐AGT‐3* Regulates Endothelial Angiogenic Effects In Vitro

**FIGURE 3:**
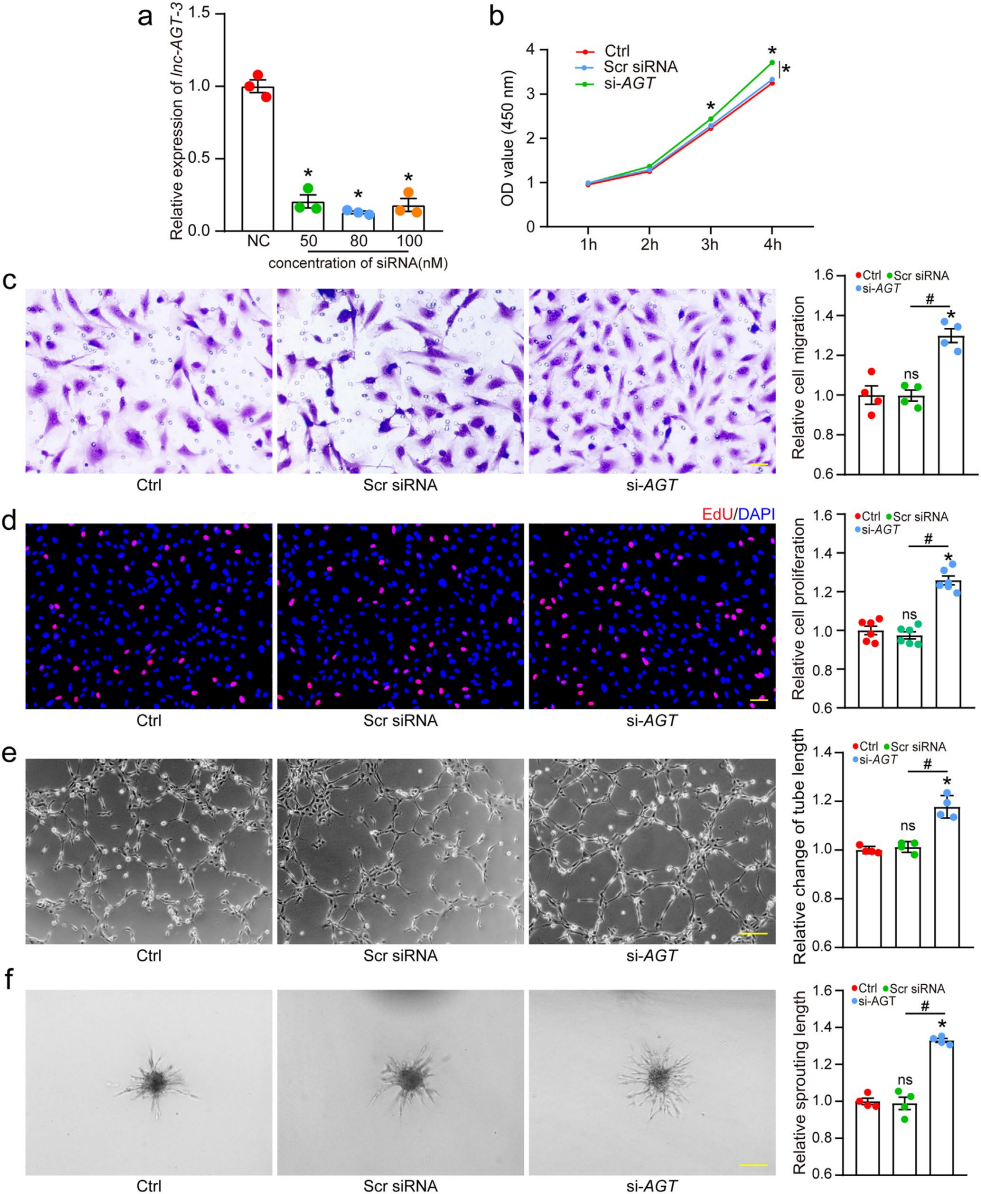
*lnc‐AGT‐3* regulates endothelial angiogenic effects in vitro. (a) RT‐PCR analysis of *lnc‐AGT‐3* expression in HUVECs after transfection with control siRNA (Scr siRNA), or *lnc‐AGT‐3* smart silencer (si‐*AGT*) (50 nM, 80 nM, 100 nM) for 24 h (*n* = 3, *p < 0.05 vs. Scr siRNA, ANOVA with Bonferroni). (b) Cell viability assessed by CCK‐8 assay after transfection (80 nM), or left untreated (Ctrl) for 24 h (*n* = 3, *p < 0.05 vs. Ctrl group; #*p* < 0.05 between the marked group; ANOVA with Bonferroni). (c–f) Functional assays after *lnc‐AGT‐3* knockdown (80 nM, 24 h). Cell migration and quantitative analysis were conducted by transwell assays (c, *n* = 4, Scale bar, 50 μm). The proliferation ability of HUVECs was determined by EdU assays (d, *n* = 6, Scale bar, 50 μm). Tube formation assays and spheroid sprouting assays were conducted to detect the tube formation ability of HUVECs (e, f, *n* = 4, Scale bar, 100 μm). *p < 0.05 versus Ctrl group; #*p* < 0.05 between the marked group; “ns” represents no statistical significance; ANOVA with Bonferroni.

Current evidence identifies endothelial cells as primary contributors to aberrant angiogenesis in ocular diseases (Li et al. 2019; Selvam et al. 2018). Therefore, we systematically investigated *lnc‐AGT‐3*'s function in endothelial cells and developed smart silencer for *lnc‐AGT‐3* knockdown in vitro. All tested concentrations of the smart silencer (50, 80, and 100 nM) effectively downregulated *lnc‐AGT‐3* expression, as validated by qRT‐PCR (Figure 3a). Firstly, we assessed the functional impact of *lnc‐AGT‐3* knockdown on HUVEC under physiological conditions. The results indicated that *lnc‐AGT‐3* silencing led to higher cell viability, enhanced migration, increased cell proliferation and tube formation (Figure 3b–e). To further investigate the role of *lnc‐AGT‐3* in endothelial sprouting, we performed spheroid sprouting assays and quantified sprouting length in vitro. Relative to the control group, *lnc‐AGT‐3* knockdown significantly increased the sprouting length of endothelial spheroids (Figure 3f). Additionally, apoptosis was assessed using propidium iodide (PI) staining and flow cytometry. We found that silencing of *lnc‐AGT‐3* significantly improved the survival of HUVECs subjected to oxidative damage induced by H_2_O_2_ (Figure S5a,b). Conversely, overexpression of *lnc‐AGT‐3* in HUVECs significantly inhibited the cells' ability of migration, proliferation, and tube formation while promoting apoptosis (Figure S6a–e). Taken together, these results demonstrated that *lnc‐AGT‐3* serves as a key modulator of endothelial cell function.

### 3.6 *lnc‐AGT‐3* Modulates Pathological Angiogenesis In Vivo and Ex Vivo

**FIGURE 4:**
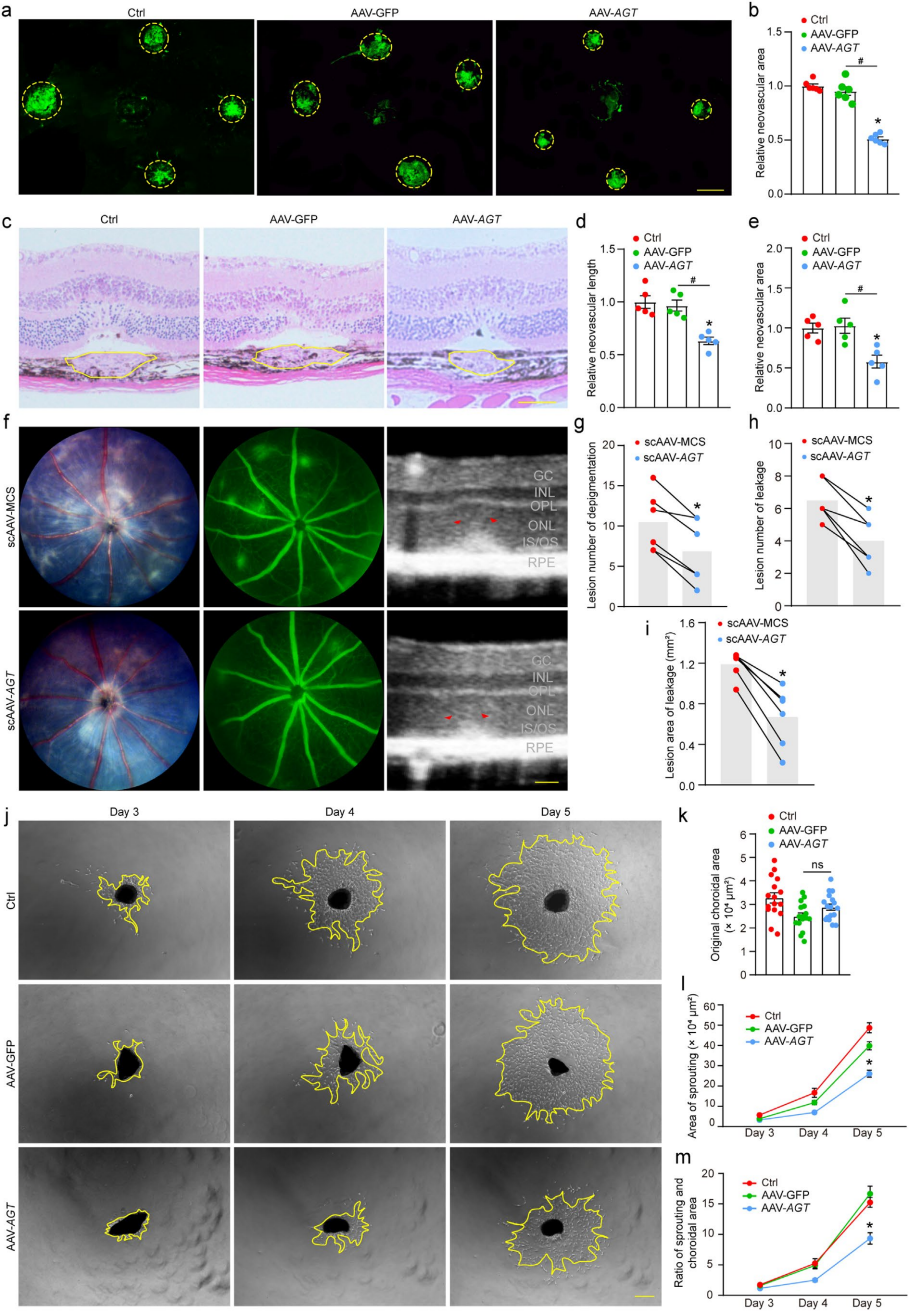
*lnc‐AGT‐3* regulates CNV development in vivo and ex vivo. (a,b) C57BL/6 mice received intravitreal injections of *lnc‐AGT‐3* overexpression AAV (2 μL, 2.0E+9), negative control AAV (AAV‐GFP), or left untreated (Ctrl). IB4‐labeled RPE/choroid flatmounts 7 days post‐laser (21 days post‐intravitreal AAV injection). Yellow circles denote CNV lesions. Four spots per eye were averaged (*n* = 6, Scale bar, 200 μm). *p < 0.05 versus Ctrl group; #*p* < 0.05 between the marked group; “ns” represents no statistical significance; ANOVA with Bonferroni. (c–e) HE‐stained retinal sections showing CNV area/length reduction with AAV‐*lnc‐AGT‐3* treatment (*n* = 5, Scale bar, 200 μm; statistical markers as above). (f–i) Fundus photographs, fluorescein angiography and OCT of NRV2 mice after intravitreal injections of scAAV‐MCS (2 μL, left eye) and scAAV‐AGT (2 μL, 2.0E+9, right eye) for 7 days (*n* = 6, Scale bar, 50 μm). The statistical results of the lesion number of depigmentation (g), lesion number of leakage (h) and lesion area of leakage (i) are shown in the figure. *p < 0.05 versus Ctrl group; paired *t*‐test/Wilcoxon test. (j–m) Choroidal sprouting assay: Representative images at Days 3–5 post‐explant (Scale bar, 200 μm); quantification of sprouting potency (*n* = 5). *p < 0.05 versus Ctrl group; ANOVA with Bonferroni.

Procedures for the animal experiments are illustrated schematically in Figure S7a. AAV was designed to overexpress *lnc‐AGT‐3* in mice. The qRT‐PCR results revealed that AAV could significantly increase *lnc‐AGT‐3* expression in the retina and choroid of mice within 1 month (Figure S7b,c). Hence, intravitreal injection of *lnc‐AGT‐3* overexpression (OE) AAV in the laser‐induced CNV model was administered to evaluate its regulatory effects on neovascularization in vivo. IB4 immunofluorescence results affirmed that *lnc‐AGT‐3* overexpression led to a reduced lesion area (Figure 4a,b). Besides, histopathological examination by H&E staining demonstrated that *lnc‐AGT‐3* overexpression decreased CNV lesion length and area (Figure 4c–e). Additionally, to meet the experimental requirements for NRV2 mice, a scAAV was designed to overexpress *lncAGT‐3* rapidly within 4 days. As before, intravitreal injection of scAAV was conducted at p18, and the results of fundus imaging and OCT were collected at p25. Compared to the negative control (scAAV‐MCS), eyes treated with scAAV‐*AGT* exhibited reduced depigmentation areas and diminished vascular leakage (Figure 4f–i). OCT imaging of the vascular leakage region revealed structural disturbances in the ONL and OPL, which were visibly ameliorated by scAAV‐*AGT* treatment (Figure 4f).

An ex vivo model of choroidal sprouting serves as a crucial tool for examining choroidal neovascularization. In this study, we assessed *lnc‐AGT‐3*'s regulatory effects on choroidal angiogenesis utilizing this ex vivo model (Shao et al. 2013). And the choroidal capillary sprouting area was quantified at sequential timepoints (Days 3–5 post‐seeding) (Figure 4j). Furthermore, the area of choroidal explants showed no significant differences in the three groups, enhancing the comparability of subsequent total sprouting area and the ratio of sprouting area analysis (Figure 4k). Quantitative analysis demonstrated that AAV‐driven *lnc‐AGT‐3* overexpression significantly reduced both the total sprouting area and the normalized sprouting ratio (sprouting area/explant area) (Figure 4l,m). Collectively, these results indicate that *lnc‐AGT‐3* overexpressing plays an anti‐angiogenic role in choridial abnormal angiogenesis.

### 3.7 *lnc‐AGT‐3* Specifically Interacts With hnRNP K

**FIGURE 5:**
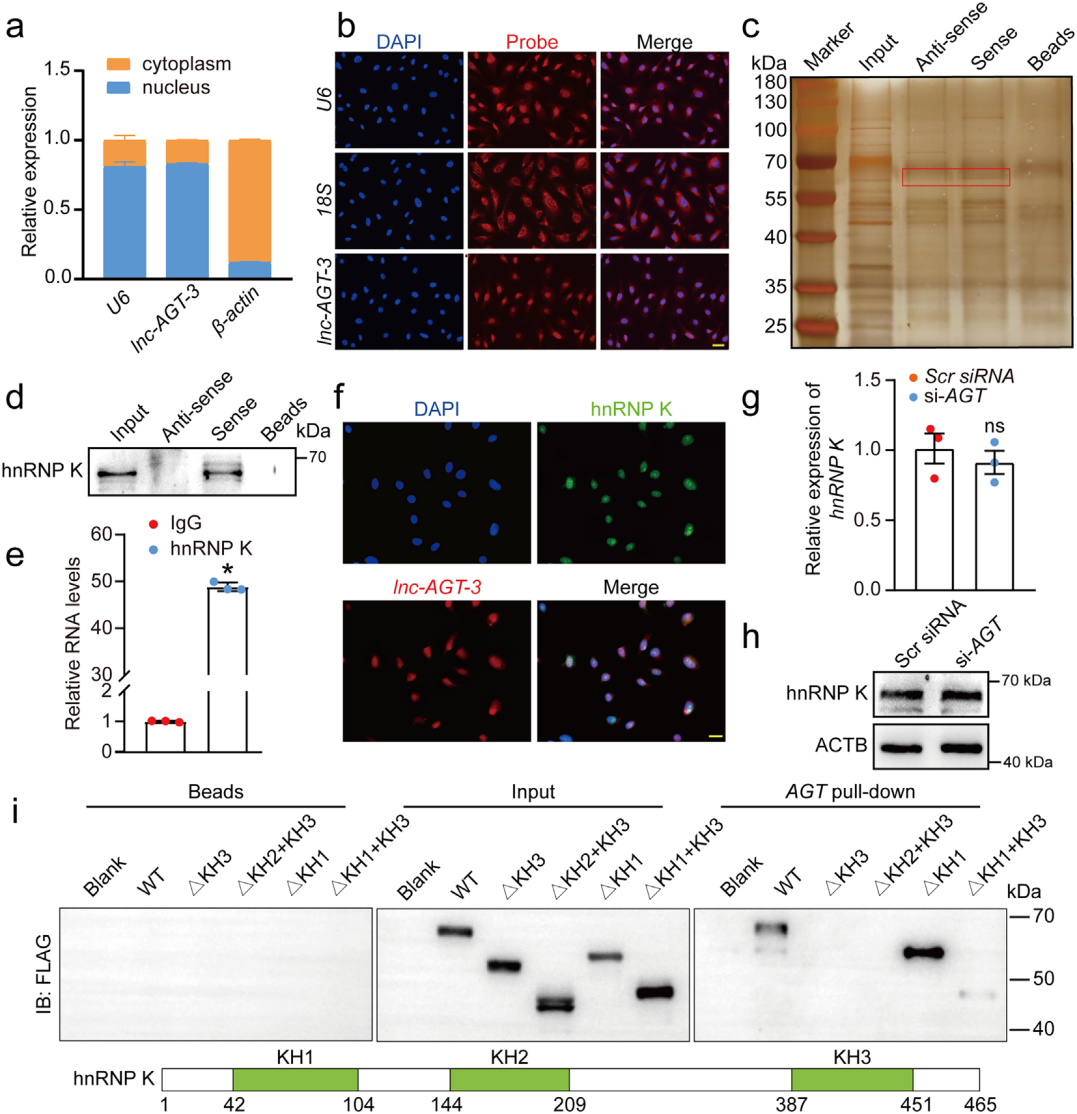
*lnc‐AGT‐3* specifically interacts with hnRNP K. (a) qRT‐PCR analysis of *lnc‐AGT‐3* distribution relative to nuclear (*U6*) and cytoplasmic (*β‐actin*) controls (*n* = 3). (b) RNA‐FISH showing *lnc‐AGT‐3* localization (Cy3, red) with nuclear (*U6*) and cytoplasmic (*18S rRNA*) controls. Nuclei counterstained with DAPI (scale bar = 20 μm). (c) *lnc‐AGT‐3*‐sense and *lnc‐AGT‐3*‐antisense RNAs were biotinylated, transcribed in vitro, and incubated with HUVEC total cell lysates for RNA pull‐down assays. After silver staining, *lnc‐AGT‐3*‐sense‐specific bands were excised and analyzed using mass spectrometry. (d) Western blot validation of hnRNP K binding to biotinylated *lnc‐AGT‐3* from pull‐down assays. (e) RIP assays using hnRNP K antibody confirming RNA‐protein interaction (*n* = 3). *p < 0.05 versus IgG; Student *t* test. (f) Co‐localization of *lnc‐AGT‐3* (red, FISH) and hnRNP K (green, IF) in HUVECs (scale bar, 20 μm). (g) The relative expression of *hnRNP K* was determined in HUVECs after *lnc‐AGT‐3* siRNA transfection by qRT‐PCR assays (*n* = 3, *p < 0.05 vs. Scr siRNA, Student *t* test). (h) The relative expression of hnRNP K was determined in HUVECs after *lnc‐AGT‐3* siRNA transfection by western blotting (*n* = 4). (i) IB assays for the flag‐tagged hnRNP Ks (wild type and various constructed truncations) were performed by in vitro–transcribed, biotinylated *lnc‐AGT‐3*.

To investigate the potential mechanisms underlying the *lnc‐AGT‐3*‐mediated anti‐angiogenic effects, we firstly probe into the sublocation of *lnc‐AGT‐3* in HUVECs by conducting sequential biochemical fractionation (separating nuclear/cytoplasmic RNA) and spatial validation via FISH. Both fractionation and FISH analyses demonstrated predominant nuclear localization of *lnc‐AGT‐3* in HUVECs (Figure 5a,b), so we speculate that *lnc‐AGT‐3* exerts its regulatory function mainly through binding proteins (Herman et al. 2022). Then, RNA pull‐down assays were performed to identify the *lnc‐AGT‐3*‐protein complex in HUVECs. Whole cell lysates from HUVECs were incubated with biotinylated *lnc‐AGT‐3* or antisense *lnc‐AGT‐3* control RNA, followed by SDS‐PAGE electrophoresis and silver staining. A prominent band (~55–70 kDa) was observed in the *lnc‐AGT‐3* probe group (Figure 5c). Consequently, the protein band was excised and subjected to mass spectrometry, which revealed hnRNP K as the most abundant protein associated with *lnc‐AGT‐3* (Table S5), so we chose this protein for subsequent experimental validation. Then, RNA pull‐down assays confirmed the direct interaction between *lnc‐AGT‐3* and hnRNP K (Figure 5d). Additionally, we verified the association of *lnc‐AGT‐3* with hnRNP K by RNA immunoprecipitation (RIP) and found that *lnc‐AGT‐3* was highly enriched in the immunoprecipitates against hnRNP K as compared with IgG (Figure 5e). After that, FISH assays and immunostaining confirmed the nuclear co‐localization of *lnc‐AGT‐3* and hnRNP K protein, further confirming the interaction between them (Figure 5f). Furthermore, we found that hnRNP K expression showed no significant changes at both transcriptional and translational levels in HUVECs lacking *lnc‐AGT‐3* (Figure 5g,h).

Extensive research has demonstrated that the K homology (KH) domains (KH1‐KH3) of hnRNP K are critical for its RNA and DNA binding capability (Wang et al. 2020). Therefore, we constructed a series of hnRNP K truncations (Flag‐tagged) to map the *lnc‐AGT‐3* binding region(s) of hnRNP K. The results showed that only the deletion of the KH3 domain abolished the interplay between hnRNPK and *lnc‐AGT‐3* compared to KH1 and KH2 domains deletion (Figure 5i), which highlights that the KH3 domain is essential for the interaction between *lnc‐AGT‐3* and hnRNP K. Collectively, these data demonstrated that *lnc‐AGT‐3* selectively interacts with the KH3 domain of hnRNP K.

### 3.8 *lnc‐AGT‐3* Exerts Anti‐Angiogenic Effects via the p53/TSP1 Axis

**FIGURE 6:**
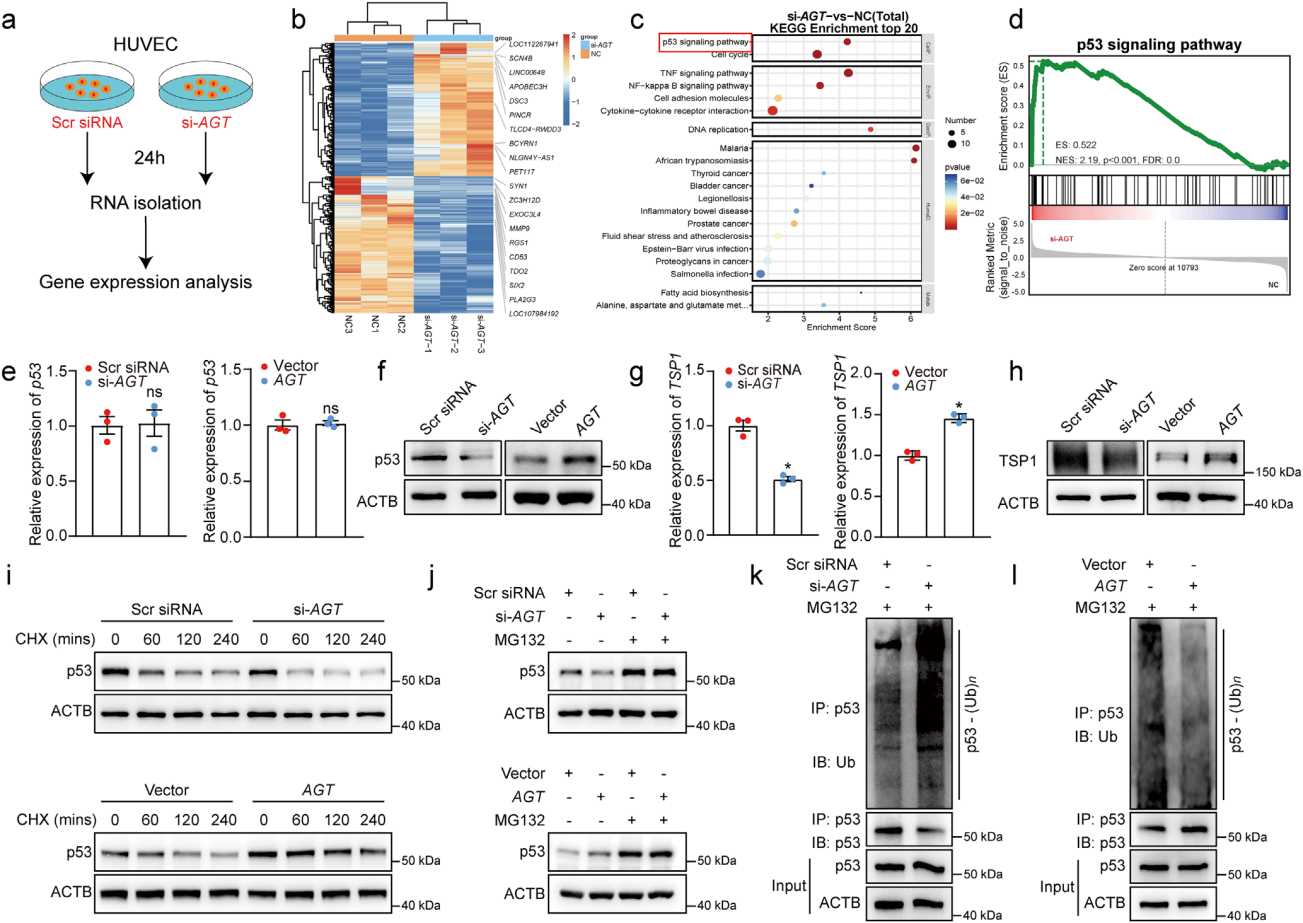
*lnc‐AGT‐3* exerts anti‐angiogenic effects via the *p53* signaling pathway. (a) HUVECs were transfected with negative control (NC) (80 nM) or *lnc‐AGT‐3 smart silencer* (80 nM) for 24 h. RNA‐seq analysis was conducted to identify differentially expressed genes following *lnc‐AGT‐3* knockdown (*n* = 3). (b) Heatmap of significantly altered genes between NC group and *lnc‐AGT‐3* silencing group according to the RNA‐seq analysis. (c) Top 20 enriched KEGG pathways from RNA‐seq data. (d) GSEA showing significant enrichment of p53 signaling pathway (si‐AGT vs. NC). (e) *lnc‐AGT‐3* siRNA (si‐*AGT*), control siRNA (Scr siRNA), *lnc‐AGT‐3* overexpression vector (*AGT*) or NC vector (Vector) was transfected into HUVECs. The relative expression of p53 was determined in HUVECs by qRT‐PCR assays (*n* = 3, “ns” represents no statistical significance, Student *t* test). (f) The relative expression of p53 was determined in HUVECs after *lnc‐AGT‐3* transfection by western blotting (*n* = 4). (g) The relative expression of *TSP1* was determined in HUVECs after *lnc‐AGT‐3* transfection by qRT‐PCR assays (*n* = 3, *p < 0.05 vs. Scr siRNA, Student *t* test). (h) The relative expression of TSP1 was determined in HUVECs after *lnc‐AGT‐3* transfection by western blotting (*n* = 4). (i) The protein levels of p53 in HUVECs with or without the treatment of cycloheximide (100 μg/mL). (j) The protein levels of p53 in HUVECs with or without the treatment of 20 μM MG132 (*n* = 4). (k, l) IB of p53 ubiquitination in HUVECs with the treatment of 20 μM MG132 (*n* = 4).

Previous studies suggest that the KH3 domain is critically involved in hnRNP K‐dependent *p53* pathway regulation (Peng et al. 2021; Qin et al. 2020). To further investigate the mechanism of *lnc‐AGT‐3*, transcriptomic analysis of HUVECs following 24‐h *lnc‐AGT‐3* silencing was conducted to identify differentially regulated downstream targets (Figure 6a). RNA‐seq analysis identified 452 differentially expressed genes (|Log2FC| ≥ 1, *p* < 0.05) upon *lnc‐AGT‐3* knockdown, with 223 upregulated and 229 downregulated transcripts relative to negative controls (NC). A heatmap was generated to illustrate the upregulated and downregulated genes (Figure 6b). To further explore the biological processes influenced by *lnc‐AGT‐3*, functional enrichment analysis (GO, KEGG, and GSEA) was conducted on the differentially expressed gene set. The results revealed significant involvement of the *p53* signaling pathway among the affected pathways (Figure 6c,d).

We next examined whether *lnc‐AGT‐3* directly regulates *p53* in HUVECs. qRT‐PCR assays revealed that neither overexpression nor knockdown of *lnc‐AGT‐3* resulted in significant changes in *p53* mRNA levels (Figure 6e). However, WB results showed that knocking down *lnc‐AGT‐3* led to a decrease in p53 protein levels. Conversely, overexpression of *lnc‐AGT‐3* promoted the accumulation of p53 protein (Figure 6f). Simultaneously, *TSP1*, an important target of *p53*, is closely associated with angiogenesis (el‐Deiry 1998; Kang et al. 2009), and therein we also have observed that knockdown of *lnc‐AGT‐3* could reduce the mRNA and protein levels of *TSP1*, while its overexpression yielded the opposite results (Figure 6g,h). Subsequently, we employed *p53* siRNA to downregulate its expression, aiming to further explore whether *lnc‐AGT‐3* mediates its anti‐angiogenic effects through a *p53*‐dependent mechanism in vitro. The cell experiments revealed that overexpressing *lnc‐AGT‐3* can inhibit cell viability, proliferation, and migration, while promoting apoptosis. Conversely, knockdown of *p53* in this context can restore these cellular functions (Figure S8a–e). Additionally, to further validate the role of *TSP1* both in vitro and in vivo, we utilized *TSP1* knockdown siRNA and AAV, respectively (Figure S9). The results revealed that *TSP1* knockdown enhanced endothelial cell function while attenuating the inhibitory effects of *lnc‐AGT‐3* overexpression on endothelial cell proliferation, migration, and tube formation (Figure S10a–f). Similarly, *TSP1* knockdown prevented the reduction in laser‐induced lesion area and choroidal sprouting area caused by AAV‐mediated overexpression of *lnc‐AGT‐3* in vivo (Figure S10g–l).

In summary, these findings indicated that *lnc‐AGT‐3* may depend on the *p53/TSP1 axis* to acquire its anti‐angiogenic function.

### 3.9 *lnc‐AGT‐3* Inhibits p53 Ubiquitination to Enhance p53 Protein Accumulation

Aforementioned results have confirmed that modulating *lnc‐AGT‐3* expression had no significant effect on *p53* mRNA levels, but could affect its protein expression. This prompted us to hypothesize that *lnc‐AGT‐3* might affect the protein degradation of p53. Then, western blot analysis was performed to assess p53 protein stability in HUVECs following cycloheximide (CHX) treatment. The findings showed that *lnc‐AGT‐3* overexpression stabilized p53 protein in HUVECs, whereas its knockdown accelerated p53 degradation (Figure 6i). Additionally, we observed that MG132, a proteasome inhibitor, could effectively attenuate the influence of *lnc‐AGT‐3* on p53 protein levels (Figure 6j), indicating the increased abundance of p53 induced by *lnc‐AGT‐3* is not attributable to transcriptional effects. Previous studies have shown that the degradation of p53 protein is predominantly mediated by the ubiquitin‐proteasome system (Pant and Lozano 2014). Therefore, to determine whether *lnc‐AGT‐3* regulates p53 ubiquitination, we treated HUVECs with MG132, performed immunoprecipitation of p53, and used the anti‐ubiquitin antibody to detect p53 ubiquitination. As a result, we found that knockdown of *lnc‐AGT‐3* increased the levels of endogenous ubiquitinated p53, while *lnc‐AGT‐3* overexpression inhibited p53 ubiquitination (Figure 6k,l). Overall, these data indicated that *lnc‐AGT‐3* functions as a molecular scaffold to block p53 ubiquitination, resulting in its nuclear accumulation and prolonged half‐life.

### 3.10 *lnc‐AGT‐3* Influences the p53 Pathway by Means of hnRNP K

**FIGURE 7:**
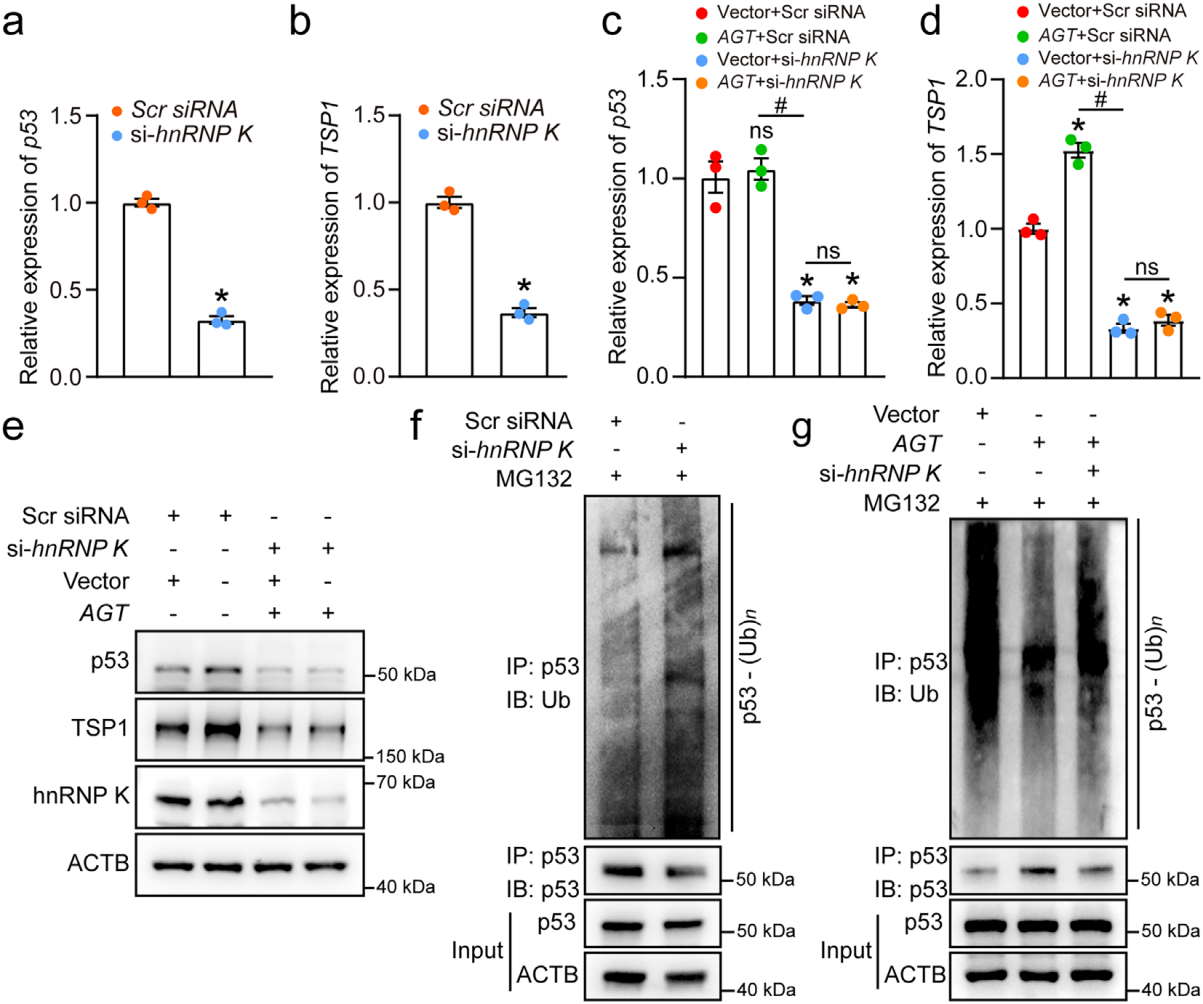
*lnc‐AGT‐3* influences the *p53* pathway by means of hnRNP K. (a, b) qRT‐PCR analysis of *p53* (a) and *TSP1* (b) mRNA levels following hnRNP K knockdown (50 nM, 24 h; *n* = 3; *p < 0.05 vs. Scr siRNA, Student *t* test). (c, d) *lnc‐AGT‐3* overexpression vector (*AGT*) or NC vector (Vector) was transfected into HUVECs. The mRNA levels of *p53* and *TSP1* in HUVECs with or without si‐*hnRNP K* (50 nM) (*n* = 3, *p < 0.05 vs. Vector + Scr siRNA group; #*p* < 0.05 between the marked group, “ns” represents no statistical significance; ANOVA with Bonferroni). (e) *lnc‐AGT‐3* overexpression vector (*AGT*) or NC vector (Vector) was transfected into HUVECs. The protein levels of p53 and TSP1 in HUVECs with or without si‐*hnRNP K* (50 nM) (*n* = 4). (f) *hnRNP K* siRNA (si‐*hnRNP K*) (50 nM) or control siRNA (Scr siRNA) (50 nM) was transfected into HUVECs. IB of p53 ubiquitination in HUVECs was conducted with the treatment of 20 μM MG132 (*n* = 4). (g) *lnc‐AGT‐3* overexpression vector (*AGT*) or NC vector (Vector) was transfected into HUVECs. IB of p53 ubiquitination in HUVECs with or without si‐*hnRNP K* (50 nM) was conducted after the treatment of 20 μM MG132 (*n* = 4).

To explore whether the p53 protein accumulation induced by *lnc‐AGT‐3* is dependent on hnRNP K, qRT‐PCR assays were firstly conducted. Interestingly, we found that *hnRNP K* knockdown significantly reduced both *p53* and *TSP1* mRNA levels (Figure S11a; Figure 7a,b). Upon *hnRNP K* knockdown, *lnc‐AGT‐3* lost its ability to modulate *p53* and downstream *TSP1* transcription, demonstrating *hnRNP K*'s essential role in this regulatory cascade (Figure 7c,d). Meanwhile, overexpression of *lnc‐AGT‐3* can promote the protein expression of p53 and TSP1, but knocking down *hnRNP K* on the basis of *lnc‐AGT‐3* overexpression can weaken this promoting effect while inhibiting the protein expression of p53 and TSP1 (Figure 7e). Consistently, *lnc‐AGT‐3* did not affect cell functions related to angiogenesis in *hnRNP K*‐depleted HUVECs (Figure S11b–e). Considering the experimental results mentioned earlier, we inferred that *lnc‐AGT‐3* might inhibit p53 ubiquitination by interacting with hnRNP K. Initially, our findings demonstrated that *hnRNP K* deletion is capable of suppressing the ubiquitination process of p53 in HUVECs (Figure 7f). Multiple studies have demonstrated that hnRNP K is a well‐characterized binding partner of p53, regulating its transcriptional function and stability (el‐Deiry 1998; Kędzierska and Piekiełko‐Witkowska 2017). To test our hypothesis, we assessed the p53 ubiquitination levels in HUVECs that had been subjected to the overexpression of *lnc‐AGT‐3* coupled with the suppression of *hnRNP K*. The results revealed that knocking down *hnRNP K* can abolish the inhibitory effect of *lnc‐AGT‐3* on p53 ubiquitination (Figure 7g). In conclusion, the data suggest that *lnc‐AGT‐3* potentially inhibits p53 ubiquitination via its interaction with hnRNP K.

## 4 Discussion

As a leading cause of irreversible vision loss globally, nAMD is currently managed primarily through anti‐VEGF interventions by inhibiting pathological angiogenesis and related complications. Nonetheless, sustained treatment is often limited by acquired resistance and potential neurotoxic effects. Here, we identified *lnc‐AGT‐3* as a highly abundant lncRNA in MSC‐exos, which was markedly upregulated upon exosomal coculture. Gain‐ and loss‐of‐function studies demonstrated that *lnc‐AGT‐3* can interact with hnRNP K, thereby inhibiting p53 ubiquitination and enhancing p53‐mediated transcriptional repression of angiogenesis. These findings establish *lnc‐AGT‐3* as an endogenous regulator of vascular homeostasis and support MSC‐exo delivery of *lnc‐AGT‐3* as a potential multi‐target strategy for overcoming limitations of current anti‐VEGF therapies in nAMD.

Building on established immunomodulatory and regenerative properties of MSC‐exos (Zhang et al. 2018; Zhou et al. 2021), our experimental evidence revealed their potent anti‐angiogenic function in neovascular eye disease. In both laser‐induced CNV and NRV2 mouse models, MSC‐exos effectively suppressed pathological angiogenesis, achieving efficacy comparable to aflibercept without compromising retinal structure or visual function. Furthermore, MSC‐exos attenuated oxidative stress (H_2_O_2_)‐induced angiogenic responses in endothelial cells and enriched the lncRNA *lnc‐AGT‐3*, which we identified as a key mediator of their anti‐angiogenic action. These findings are consistent with prior reports of exosomal cargo delivery (Huang et al. 2020; Pan et al. 2024), providing a translational framework supporting MSC‐exos as a promising therapeutic alternative, particularly for patients with suboptimal responses to current anti‐VEGF therapies.

MSC‐exosomes carry a multifaceted repertoire of non‐coding RNAs and effector proteins capable of reprogramming cellular activities in recipient cells. The RNAs, including coding and noncoding RNAs (ncRNAs), play crucial roles in a broad range of physiological and pathological processes. In contrast to protein‐coding RNAs, ncRNAs critically regulate cellular functions including chromatin remodeling, RNA splicing, and mRNA stability (Ransohoff et al. 2018; Tan et al. 2021). Numerous studies have highlighted that lncRNAs are capable of orchestrating the proliferation‐apoptosis balance, cell differentiation, and therapeutic responses through diverse molecular mechanisms. For example, we previously identified lncRNA *ZNF503‐AS1* as a positive regulator of RPE differentiation via specific gene regulatory networks (Chen et al. 2017). Increasing studies indicate that lncRNAs, particularly when encapsulated within exosomes, play pivotal roles in normal homeostasis and disease progression. For instance, Cao et al. reported that exosomal SNHG7 from MSCs inhibits endothelial‐mesenchymal transition and angiogenesis in diabetic retinopathy by targeting the *miR‐34a‐5p*/XBP1 pathway (Cao et al. 2021). Mechanistically, the lncRNAs achieve their functions through diverse mechanisms, including scaffolding protein assemblies, directing chromatin modifiers to target loci, and acting as miRNA sponges to regulate gene expression (Ransohoff et al. 2018). However, the precise intracellular mechanisms of exosomal lncRNAs in nAMD pathogenesis and their systemic regulatory networks remain elusive, necessitating further mechanistic exploration.

Through microarray analysis, we identified *lnc‐AGT‐3* as a novel angiogenesis regulator that is highly enriched in MSC‐exosomes but markedly downregulated in nAMD pathogenesis. The restoration of *lnc‐AGT‐3* levels through MSC‐exosomes or AAV‐mediated overexpression effectively attenuated CNV and improved retinal structure across in vivo and ex vivo models, with complementary in vitro validation demonstrating its anti‐angiogenic activity in HUVECs. Specifically, *lnc‐AGT‐3* silencing augmented key angiogenic processes (migration, proliferation, tube formation), whereas its overexpression resulted in the reverse outcomes, collectively demonstrating its role as an endogenous brake on endothelial cell function and pathological neovascularization.

We further elucidated the underlying mechanisms through which *lnc‐AGT‐3* regulates ocular angiogenesis. lncRNAs located in the nucleus frequently function as molecular scaffolds, facilitating interactions with RNA‐binding proteins (RBPs). Given that *lnc‐AGT‐3* is predominantly localized in the nucleus in our study, RNA pull‐down assays were performed to identify *lnc‐AGT‐3*‐interacting RBPs. The results demonstrated that *lnc‐AGT‐3* specifically binds to hnRNP K, a well‐characterized nuclear protein that is widely recognized for its interactions with various lncRNAs. Evidence has shown that hnRNP K serves as a critical functional partner for numerous lncRNAs. For example, hnRNP K cooperates with *LINC00571* to regulate tricarboxylic acid cycle metabolites, leading to the progression of triple‐negative breast cancer (Xi et al. 2024). *lncRNA MGCG* binds to hnRNP K to promote tumor development and autophagy in glioblastoma (Chu et al. 2023). The structure and function of hnRNP K, with its three KH domains and a unique KI domain, enable it to interact with both DNA and RNA, as well as to modulate gene expression at multiple levels (Zhu et al. 2019). Moreover, the RNA‐binding KH3 motif of hnRNP K was identified as the critical region for its interaction with *lnc‐AGT‐3*. This interaction highlights the potential role of *lnc‐AGT‐3* in modulating nuclear processes through its association with hnRNP K.

To further investigate the potential downstream signaling pathway, we conducted RNA‐seq analysis and demonstrated that *lnc‐AGT‐3* mediates its anti‐angiogenic activity by coordinately activating p53 transcriptional targets. Analogously, a recent study found that MSC‐exos could activate the *p53* pathway through targeted delivery of internal microRNAs (Li et al. 2024). Furthermore, plenty of studies have underscored the critical involvement of the *p53* signaling pathway in regulating tumor angiogenesis, emphasizing its pivotal role in modulating angiogenic processes (Cao et al. 2020; Song et al. 2015). Additionanlly, recent advances have revealed that lncRNAs are involved in regulating p53 activity via a variety of molecular mechanisms (Mao et al. 2018; Yuan et al. 2022). Here, we observed that *lnc‐AGT‐3* overexpression stabilizes p53 protein and upregulates its downstream effector *TSP1*, which mediates potent anti‐angiogenic effects in pathological neovascularization (Kang et al. 2009; Sundaram et al. 2011). Nevertheless, the molecular mechanisms by which lncRNAs regulate p53 stability via post‐translational modifications remain poorly characterized and require systematic investigation. In this study, we have demonstrated that *lnc‐AGT‐3* could influence the protein levels of p53, an effect likely mediated by its direct binding to hnRNP K. Notably, *lnc‐AGT‐3* does not affect p53 expression at the mRNA level. However, our findings reveal that silencing hnRNP K alone results in a reduction of p53 mRNA levels. This suggests that hnRNP K can regulate p53 expression through additional mechanisms beyond its interaction with *lnc‐AGT‐3*. The specific interaction between *lnc‐AGT‐3* and hnRNP K, which inhibits p53 ubiquitination, represents only one of the pathways through which hnRNP K modulates p53. Furthermore, multiple studies have corroborated that hnRNP K can influence chromatin remodeling and transcription (Bomsztyk et al. 2004; Ostrowski et al. 2003). Despite these findings, our conclusion that *lnc‐AGT‐3* contributes to the regulation of the p53 pathway via its interaction with hnRNP K, and thereby plays a role in the pathogenesis of nAMD, remains supported. Future research should aim to further elucidate the precise molecular mechanisms by which hnRNP K regulates p53 expression and functional activity. Taken together, these observations underscore the role of *lnc‐AGT‐3* in regulating p53 stability, offering a significant addition to the complex regulatory network governing p53 activity.

Beyond revealing key mechanistic insights into nAMD, our findings provide the foundation for novel therapeutic strategies. The ability of MSC‐exosomes to precisely target pathogenic pathways underscores their potential as a targeted, multi‐modal treatment platform, which may help address current limitations in the management of this vision‐threatening disease. However, several challenges remain to be addressed. While the laser‐induced CNV model served as a robust platform for initial mechanistic validation, future studies employing aged animals or models of sustained VEGF expression will be valuable to assess the efficacy of MSC‐exosomes in settings of treatment resistance or chronic disease activity. Moreover, the clinical correlation analysis was based on a relatively small cohort (12 nAMD patients vs. 12 controls), and the observed differential expression of *lnc‐AGT‐3* in aqueous humor, though statistically significant, requires validation in larger, multi‐center populations. Additionally, the molecular crosstalk between MSC‐exosomes and recipient cells in the complex microenvironment of neovascular lesions requires further elucidation. These limitations underscore the necessity of rigorous mechanistic studies and prospective clinical trials to determine whether the promising preclinical effects documented here can be successfully translated into human therapies. Nonetheless, our work provides a compelling mechanistic rationale and experimental framework to justify such future investigations.

Collectively, our findings establish *lnc‐AGT‐3* as a key functional cargo of MSC‐exos that attenuates nAMD‐associated angiogenesis by stabilizing p53 protein via hnRNP K binding. This work reveals a previously unrecognized mechanism by which lncRNAs can modulate ocular neovascularization through precise regulation of the p53 signaling pathway and provides a conceptual foundation for the development of targeted, RNA‐based therapeutic strategies for nAMD.

## Author Contributions

C.Z. designed the study. L.K., X.H., and S.Q. conducted the experiments and acquired data. L.K., X.H., and C.Z. wrote the manuscript. D.L., J.Z., and L.Z. developed the methods and data analysis. B.Y., Q.J., and S.Z. contributed to the critical discussion of results. All authors critically reviewed, edited, and approved the final manuscript. C.Z. is the guarantor of this work and, as such, had full access to all the data in the study and takes responsibility for the integrity of the data and the accuracy of the data analysis.

## Funding

This work was supported by the National Natural Science Foundation of China (82530031, 82020108006 to C.Z., 82501299 to S.Q.); the Open Research Funds of the State Key Laboratory of Ophthalmology, Zhongshan Ophthalmic Center, Sun Yat‐sen University.

## Conflicts of Interest

The authors declare no conflicts of interest.

## Supporting information


**Figure S1:** Uptake of exosomes by ocular tissues.
**Figure S2:** Delivery of MSC‐exos has no obvious retinal toxicity in vivo.
**Figure S3:** MSC‐exos did not influence the retinal morphology and vision function in normal mice eyes.
**Figure S4:** MSC‐exos regulates endothelial angiogenic effects in vitro.
**Figure S5:** *lnc‐AGT‐3* regulates endothelial angiogenic effects in vitro.
**Figure S6:** *lnc‐AGT‐3* regulates endothelial angiogenic effects in vitro.
**Figure S7:** The efficiency of overexpression AAV in mice.
**Figure S8:** Knockdown of p53 on the basis of *lnc‐AGT‐3* overexpression can restore cellular functions.
**Figure S9:** Verification of knockdown efficiency of TSP1 in vitro and in vivo.
**Figure S10:** TSP1 mediates *lnc‐AGT‐3*‐driven anti‐angiogenic effects in vitro and in vivo.
**Figure S11:** hnRNP K mediates *lnc‐AGT‐3*‐driven HUVEC proliferation, migration, tube formation and apoptosis.
**Table S1:** The information of ARC patients and nAMD patients involved in the study.
**Table S2:** Target sequences of *lnc‐AGT‐3* smart silencer.
**Table S3:** All RNAi Sequences used in this study.
**Table S4:** Primer sequences of *lnc‐AGT‐3* used in RNA‐Pulldown.
**Table S5:** Top 5 proteins interacting with *lnc‐AGT‐3* identified by mass spectrometry.
**Table S6:** Primer sequences used for qPCR assays.

## Data Availability

The data that support the findings of this study are available on request from the corresponding author.
