# Supplementary material for: MSC‐Derived Exosomal lnc‐AGT‐3: A Novel Anti‐Angiogenic Target in Age‐Related Macular Degeneration Through p53 Signaling Pathway
Source: Aging Cell. 2026 Jan 12;25(2):e70377. doi: 10.1111/acel.70377 (PMC12794283; doi:10.1111/acel.70377)
Supplement: Supplementary file 1 — Figure S1: Uptake of exosomes by ocular tissues. Figure S2: Delivery of MSC‐exos has no obvious retinal toxicity in vivo. Figure S3: MSC‐exos did not influence the retinal morphology and vision function in normal mice eyes. Figure S4: MSC‐exos regulates endothelial angiogenic effects in vitro. Figure S5: lnc‐AGT‐3 regulates endothelial angiogenic effects in vitro. Figure S6: lnc‐AGT‐3 regulates endothelial angiogenic effects in vitro. Figure S7: The efficiency of overexpression AAV in mice. Figure S8: Knockdown of p53 on the basis of lnc‐AGT‐3 overexpression can restore cellular functions. Figure S9: Verification of knockdown efficiency of TSP1 in vitro and in vivo. Figure S10: TSP1 mediates lnc‐AGT‐3‐driven anti‐angiogenic effects in vitro and in vivo. Figure S11: hnRNP K mediates lnc‐AGT‐3‐driven HUVEC proliferation, migration, tube formation and apoptosis. Table S1: The information of ARC patients and nAMD patients involved in the study. Table S2: Target sequences of lnc‐AGT‐3 smart silencer. Table S3: All RNAi Sequences used in this study. Table S4: Primer sequences of lnc‐AGT‐3 used in RNA‐Pulldown. Table S5: Top 5 proteins interacting with lnc‐AGT‐3 identified by mass spectrometry. Table S6: Primer sequences used for qPCR assays. [file ACEL-25-e70377-s001.docx]

**Supplemental Information**

**MSC-derived exosomal *lnc-AGT-3*: a game-changer for treating neovascular age-related macular degeneration.**

Lingjie Kong,^1,2,3#^ Xiaoyan Han,^1,2,3#^ Siyi Qi,^1,2,3#^ Duo Li,^5^ Jingyue Zhang,^5^ Linyu Zhang,^5^ Shujie Zhang,^1,2,3^ Qin Jiang,^5*^ Biao Yan,^6*^ Chen Zhao^1,2,3,4*^

Contents:

- Supplementary Figure 1. Uptake of exosomes by ocular tissues.
- Supplementary Figure 2. Delivery of MSC-exos has no obvious retinal toxicity in vivo.
- Supplementary Figure 3. MSC-exos did not influence the retinal morphology and vision function in normal mice eyes.
- Supplementary Figure 4. MSC-exos regulates endothelial angiogenic effects in vitro.
- Supplementary Figure 5. lnc-AGT-3 regulates endothelial angiogenic effects in vitro. Supplementary Figure 6. lnc-AGT-3 regulates endothelial angiogenic effects in vitro.
- Supplementary Figure 7. The efficiency of overexpression AAV in mice.
- Supplementary Figure 8. Knockdown of p53 on the basis of lnc-AGT-3 overexpression can restore cellular functions.
- Supplementary Figure 9. Verification of knockdown efficiency of TSP1 in vitro and in vivo.
- Supplementary Figure 10. TSP1 mediates lnc-AGT-3-driven anti-angiogenic effects in vitro and in vivo.
- Supplementary Figure 11. hnRNP K mediates lnc-AGT-3-driven HUVEC proliferation, migration, tube formation and apoptosis.
- Supplemental materials and methods
- Table S1: The information of ARC patients and nAMD patients involved in the study
- Table S2: Target sequences of lnc-AGT-3 smart silencer
- Table S3: All RNAi Sequences used in this study
- Table S4: Primer sequences of lnc-AGT-3 used in RNA-Pulldown
- Table S5: Top 5 proteins interacting with lnc-AGT-3 identified by mass spectrometry
- Table S6: Primer sequences used for qPCR assays

**
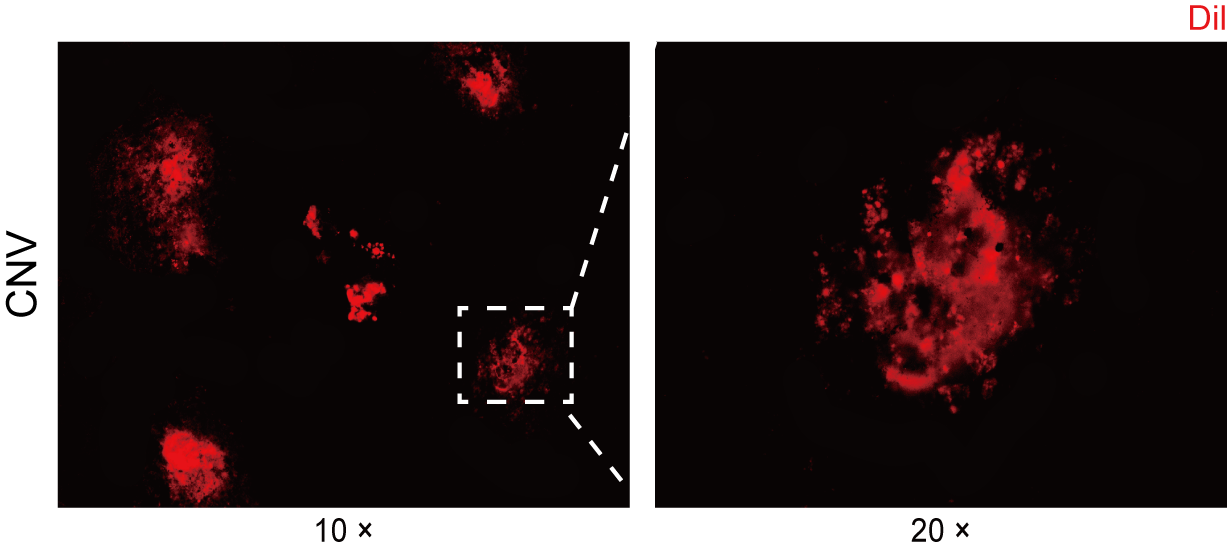
**

**Supplementary Figure 1. Uptake of exosomes by ocular tissues.**

Dil-labeled exosomes (red) in CNV lesions at 10× (Left) and 20× (right) magnification.**
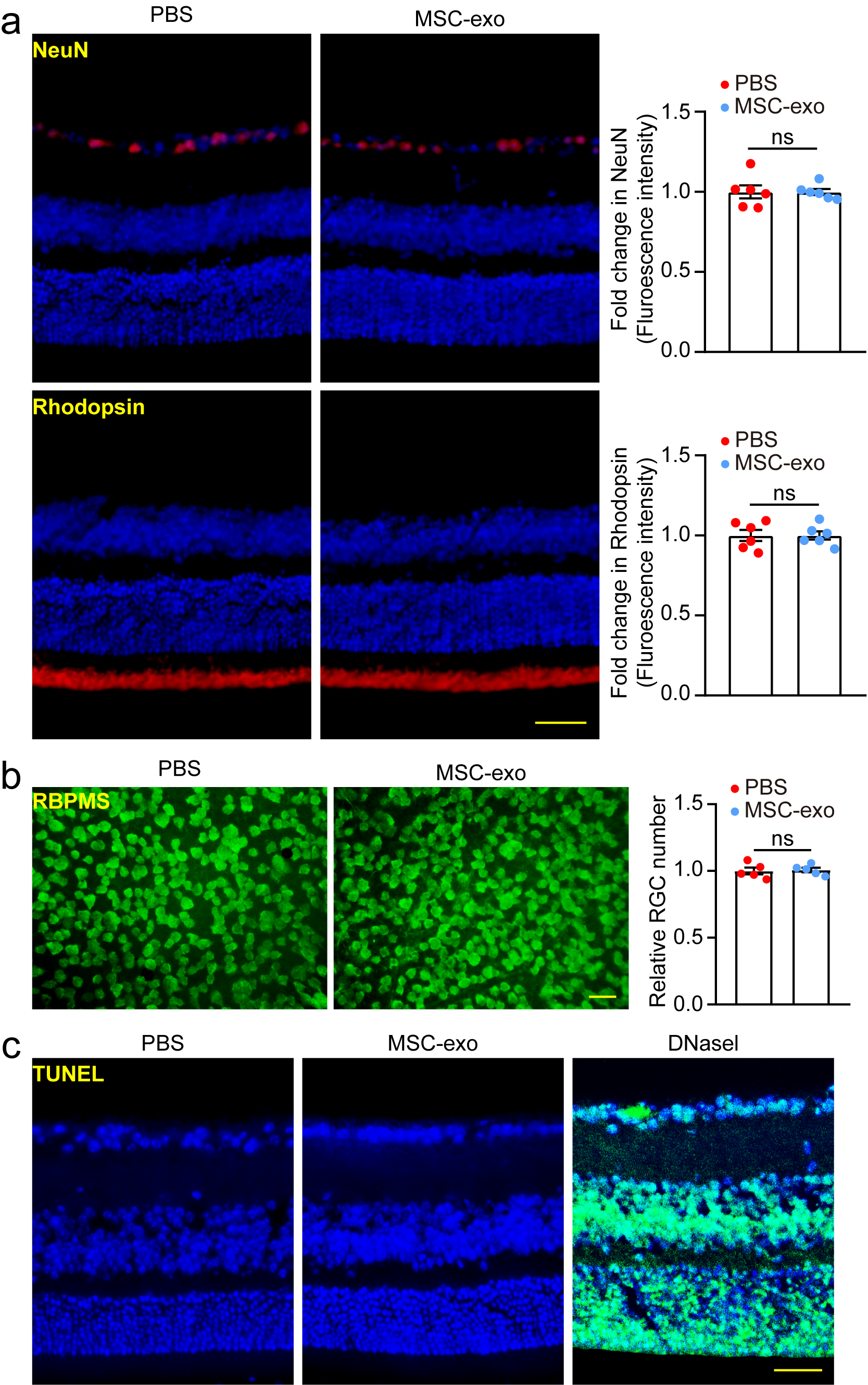
**

**Supplementary Figure 2. Delivery of MSC-exos has no obvious retinal toxicity in vivo.**

a. Immunofluorescence staining of the retinas injected with PBS (2 μL) or MSC-exos (2 μL, 50 μg) for 30 days with NeuN and Rhodopsin (Scale bar, 50 μm). Quantification results and representative images of NeuN and Rhodopsin staining were shown (n = 6). b. Retinal ganglion cell (RBPMS) staining in mid-peripheral retina 30 days post-treatment (Scale bar, 20 μm). Quantitative results and representative images of RBPMS staining are depicted (n = 5). The displayed images were captured at a location halfway between the center and the periphery of retina. c. TUNEL staining of the retinas injected with PBS (2 μL) or MSC-exos (2 μL, 50 μg) for 30 days (Scale bar, 50 μm). “ns” represents no statistical significance; Student *t* test.

**
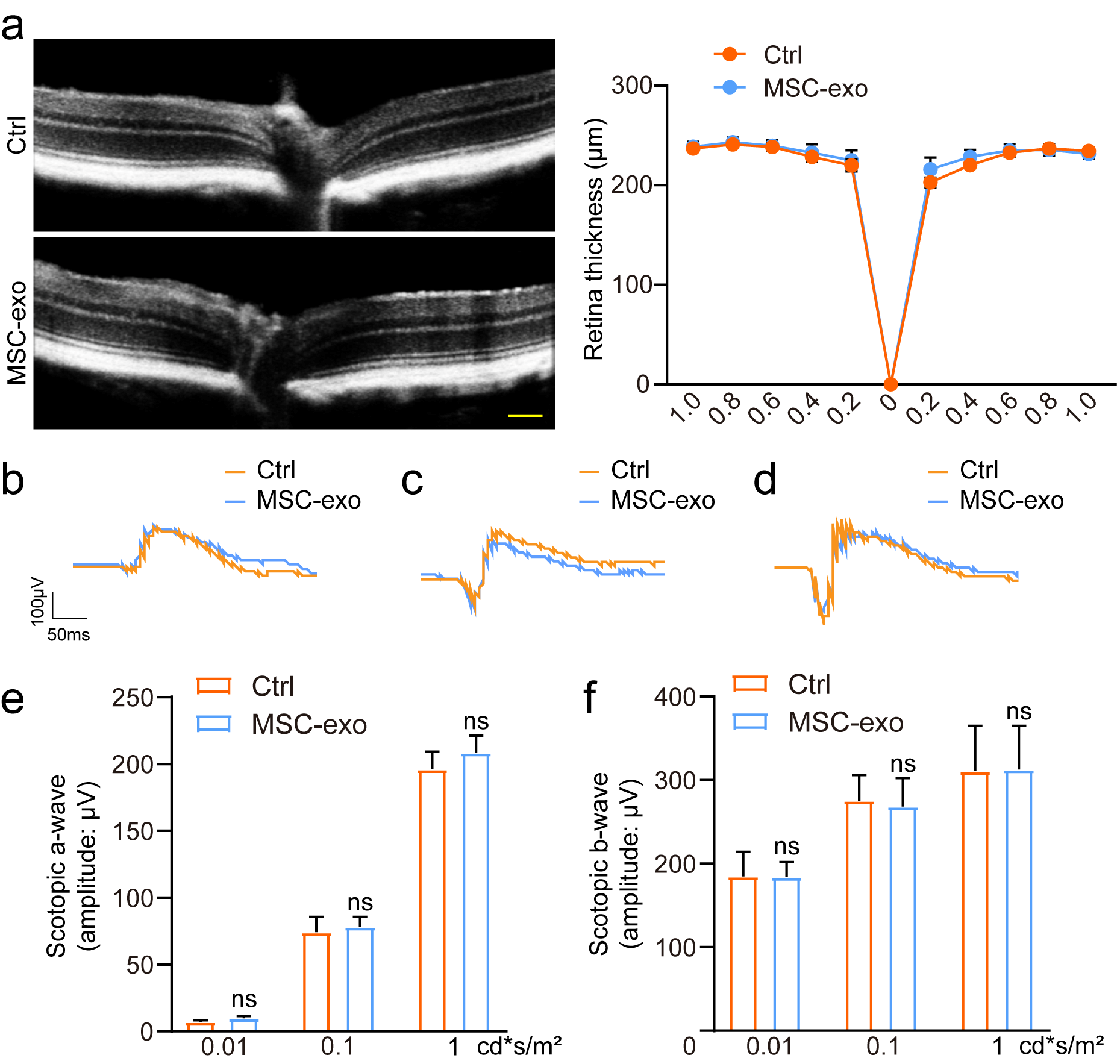
**

**Supplementary Figure 3. MSC-exos did not influence the retinal morphology and vision function in normal mice eyes.**

a. Retinal thickness analysis showing no significant changes following MSC-exosome administration (n = 10, Scale bar, 100 μm). b-d. Representative electroretinography (ERG) wave front on 24 h dark adapted mice showed alterations in both (e) scotopic a- and (f) b-wave responses measured at three different light intensities (0.01, 0.1 and 1 cd.s/m2) (n = 10, “ns” represents no statistical significance, Student *t* test).

**
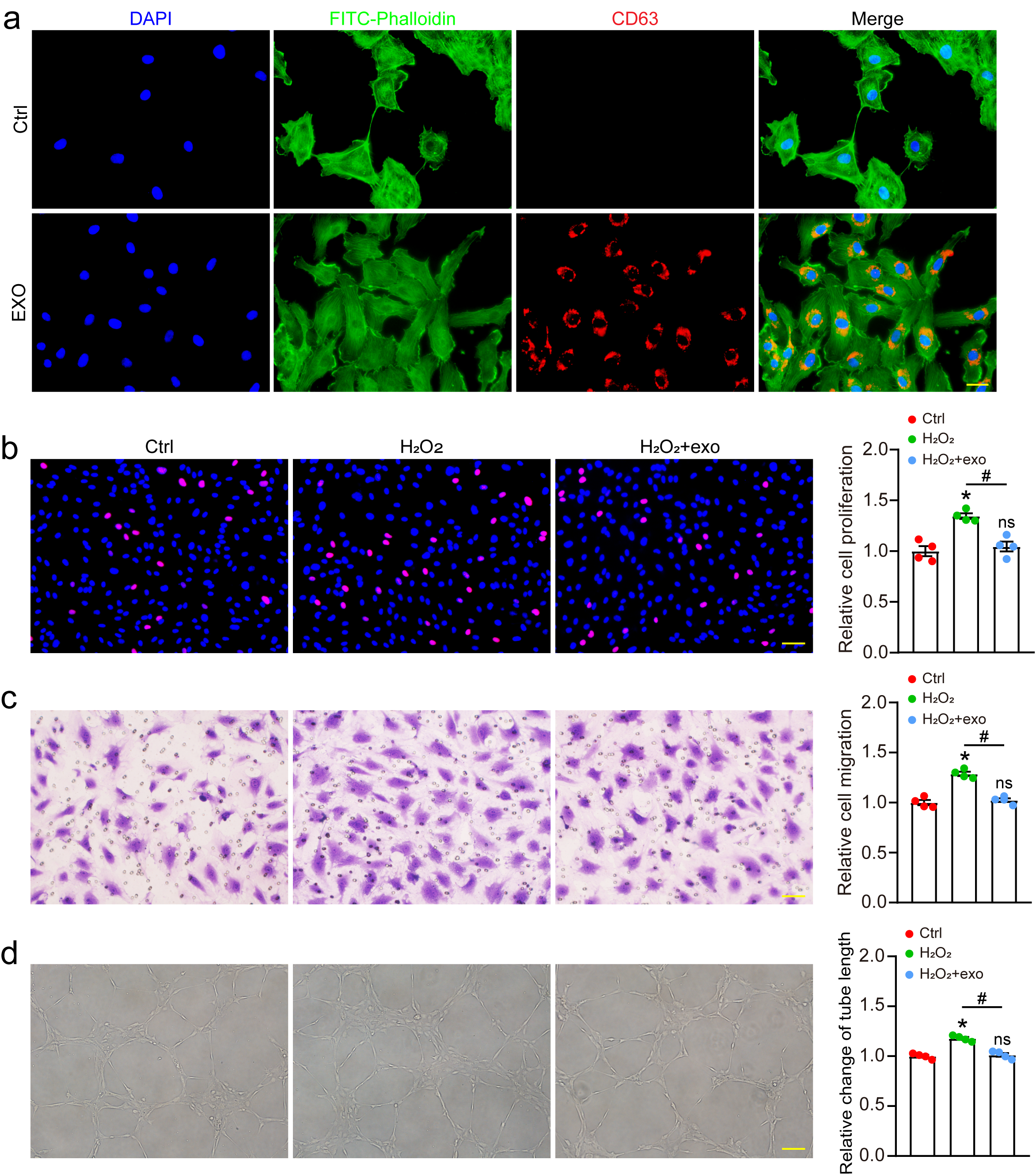
**

**Supplementary Figure 4. MSC-exos regulates endothelial angiogenic effects *in vitro*.**

a. MSC-exos were firstly labeled with anti-CD63, and then cocultured with HUVECs for 24 h. Cells were fixed and incubated with fluorescent secondary antibody. F-actin were labeled by Phalloidin (green). Scale bar, 20 μm. b-d. HUVECs were treated with H_2_O_2_ (5 μM) for 24 h, then the cells were cocultured with MSC-exos (H_2_O_2_+exo) or not (H_2_O_2_) for another 24 h. The untreated group were used as a control (Ctrl). The proliferation ability of HUVECs was determined by EdU assays (b, n = 4, Scale bar, 50 μm). Cell migration and quantitative analysis was conducted by transwell assays (c, n = 4, Scale bar, 50 μm). Tube formation assays were conducted to detect the tube formation ability of HUVECs (d, n = 4, Scale bar, 100 μm). **P* < 0.05 vs. Ctrl group; #*P* < 0.05 between the marked group; “ns” represents no statistical significance; One-way ANOVA followed by Bonferroni’s post hoc test.

**
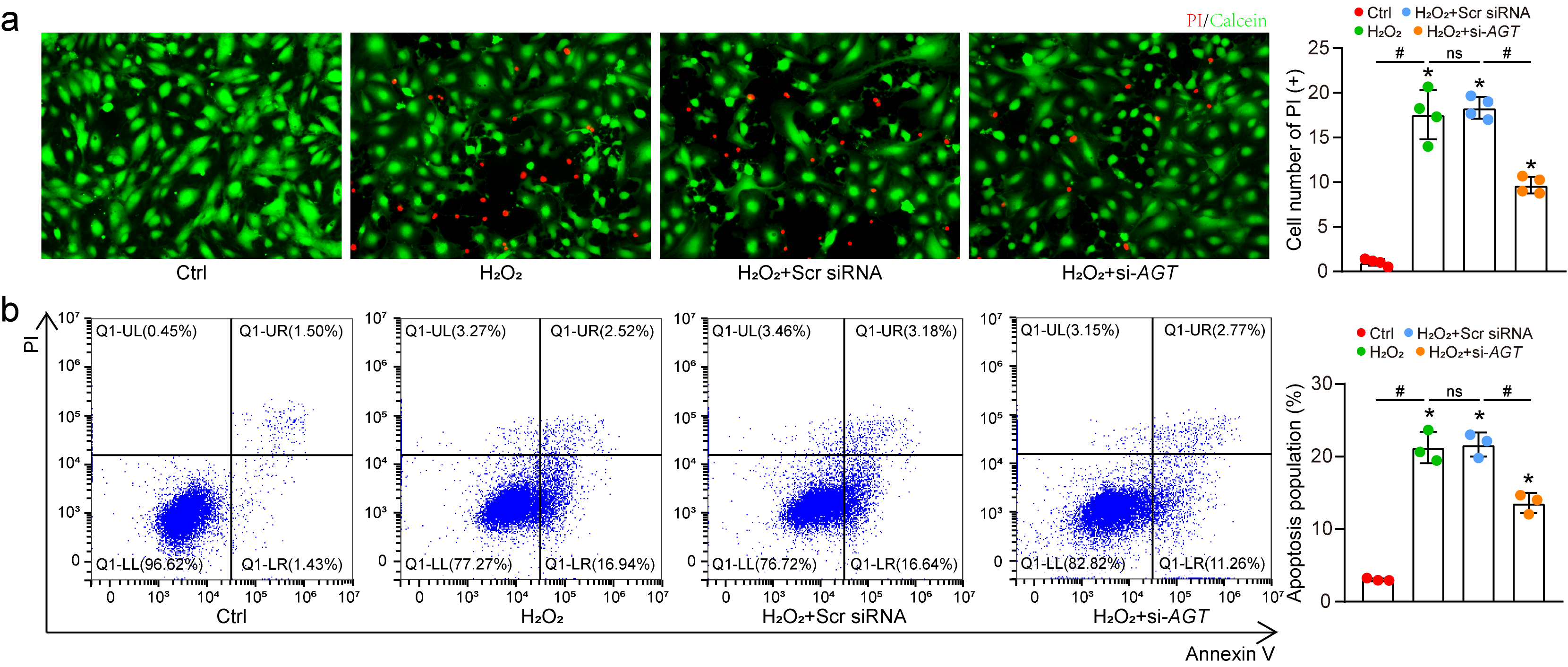
**

**Supplementary Figure 5. *lnc-AGT-3* regulates endothelial angiogenic effects *in vitro*.**

HUVECs were transfected with Scr siRNA (80 nM), *lnc-AGT-3* smart silencer (80 nM), or left untreated (Ctrl) for 24 h, and then treated with or without H_2_O_2_ (300 μM) for 24 h. Calcein-AM/PI assays (a) and flow cytometry (b) were conducted to detect cell apoptosis (a, n = 4, Scale bar, 50 μm). **P* < 0.05 vs. Ctrl group; #*P* < 0.05 between the marked group; “ns” represents no statistical significance; One-way ANOVA followed by Bonferroni’s post hoc test.

**
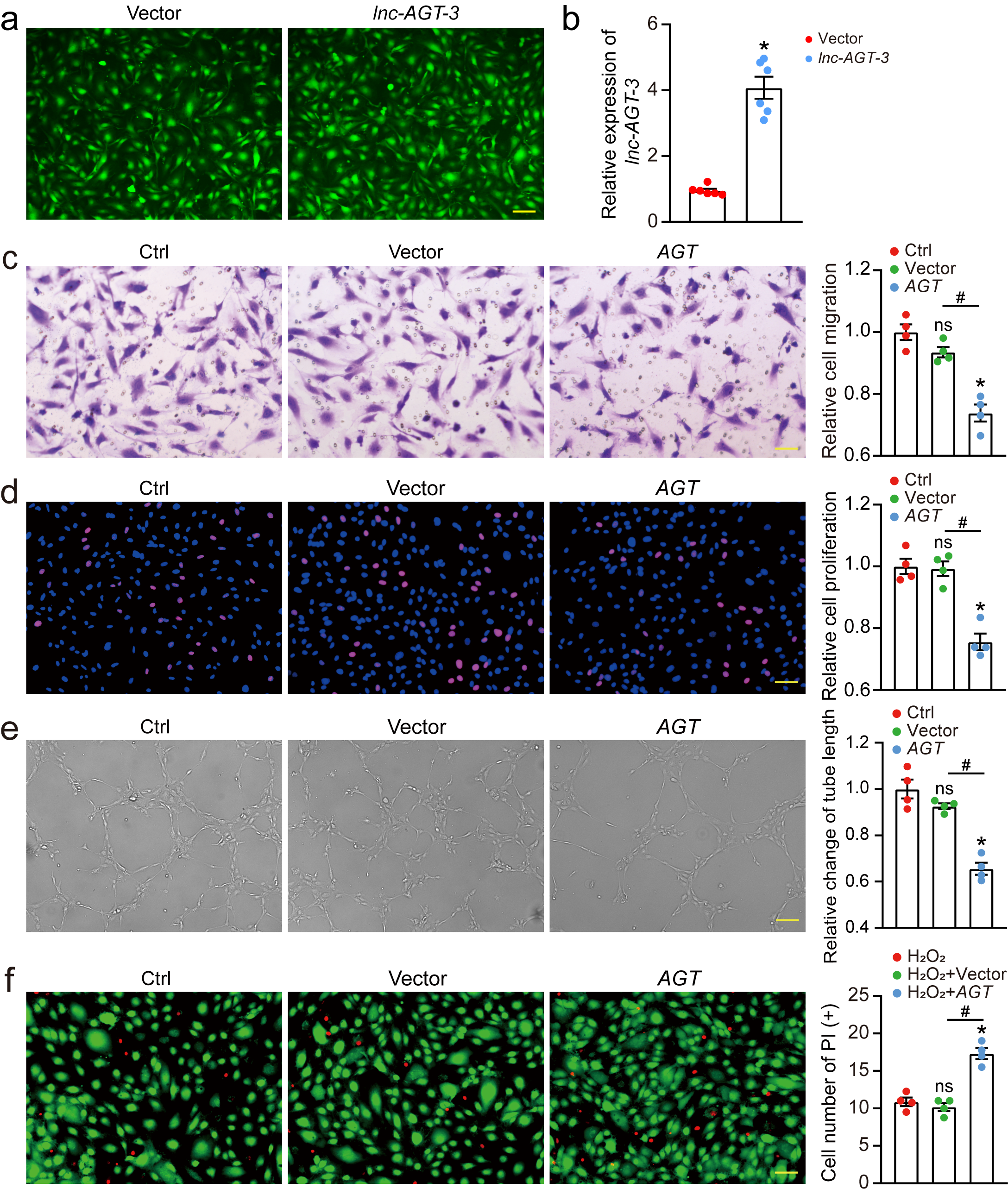
**

**Supplementary Figure 6. *lnc-AGT-*3 regulates endothelial angiogenic effects *in vitro*.**

*lnc-AGT-3* stable overexpression vector (*lnc-AGT-3*) or NC vectors (Vector) were transfected into HUVECs. a. Fluorescence photography of HUVECs transfected with lnc-AGT-3 overexpression lentivirus or vector (Scale bar, 50 μm). b. The relative expression of *lnc-AGT-3* was determined in HUVECs after *lnc-AGT-3* lentivirus transfection by qRT-PCR assays (n = 6, **P* < 0.05 vs. Vector, Student *t* test). c-e. Cell migration and quantitative analysis was conducted by transwell assays (c, n = 4, Scale bar, 50 μm). The proliferation ability of HUVECs was determined by EdU assays (d, n = 4, Scale bar, 50 μm). Tube formation assays were conducted to detect the tube formation ability of HUVECs (e, n = 4, Scale bar, 100 μm). **P* < 0.05 vs. Ctrl group; #*P* < 0.05 between the marked group; “ns” represents no statistical significance; One-way ANOVA followed by Bonferroni’s post hoc test. f. After stimulating by H_2_O_2_ (300 μM) for 24 h, calcein-AM/PI assays were conducted to detect cell apoptosis (n = 4, Scale bar, 50 μm). **P* < 0.05 vs. H_2_O_2_ group; #*P* < 0.05 between the marked group; “ns” represents no statistical significance; One-way ANOVA followed by Bonferroni’s post hoc test.

**
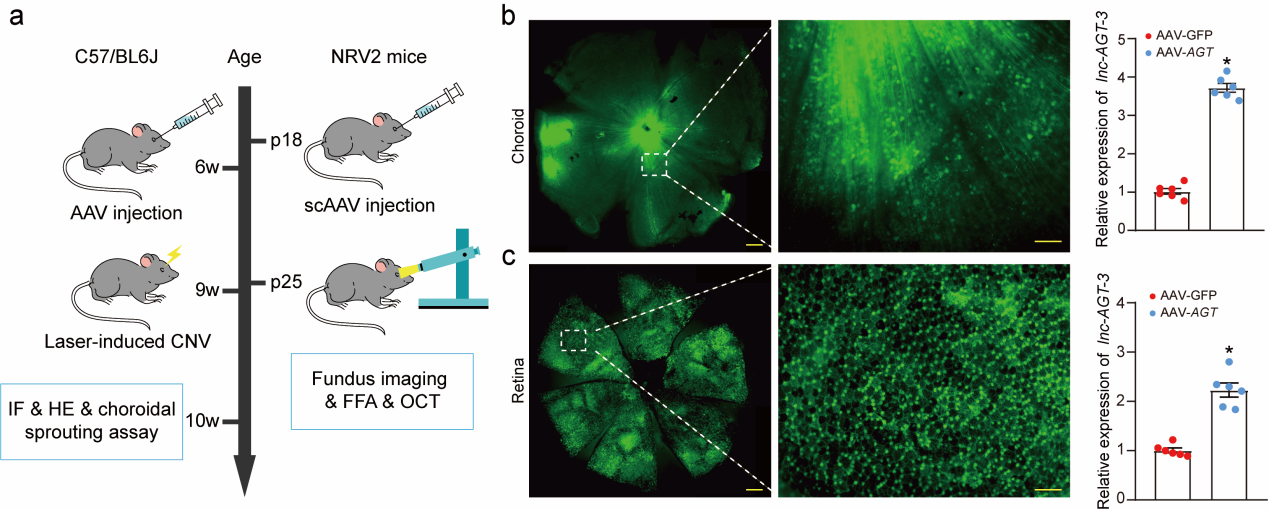
**

**Supplementary Figure 7. The efficiency of overexpression AAV in mice.**

a. Diagram illustrating the experimental procedure for assessing the anti-angiogenic effects of *lnc-AGT-*3 on laser-induced CNV mice and NRV2 mice (*Crb1^rd8^Jak3^m1J^*/Boc). b-c. C57BL/6 mice received intravitreal injections of *lnc-AGT-3* overexpression AAV (2 μL, 2.0E+9) for 1 month. The mice were euthanized, then the retina and RPE/choroid complexes were dissected and flat-mounted for fluorescence observation (green) (Scale bar, 200 μm). The levels of *lnc-AGT-*3 expression in retina and choroid were detected by qRT-PCR assays (n = 6, **P* < 0.05 vs. AAV-GFP, Student *t* test).

**
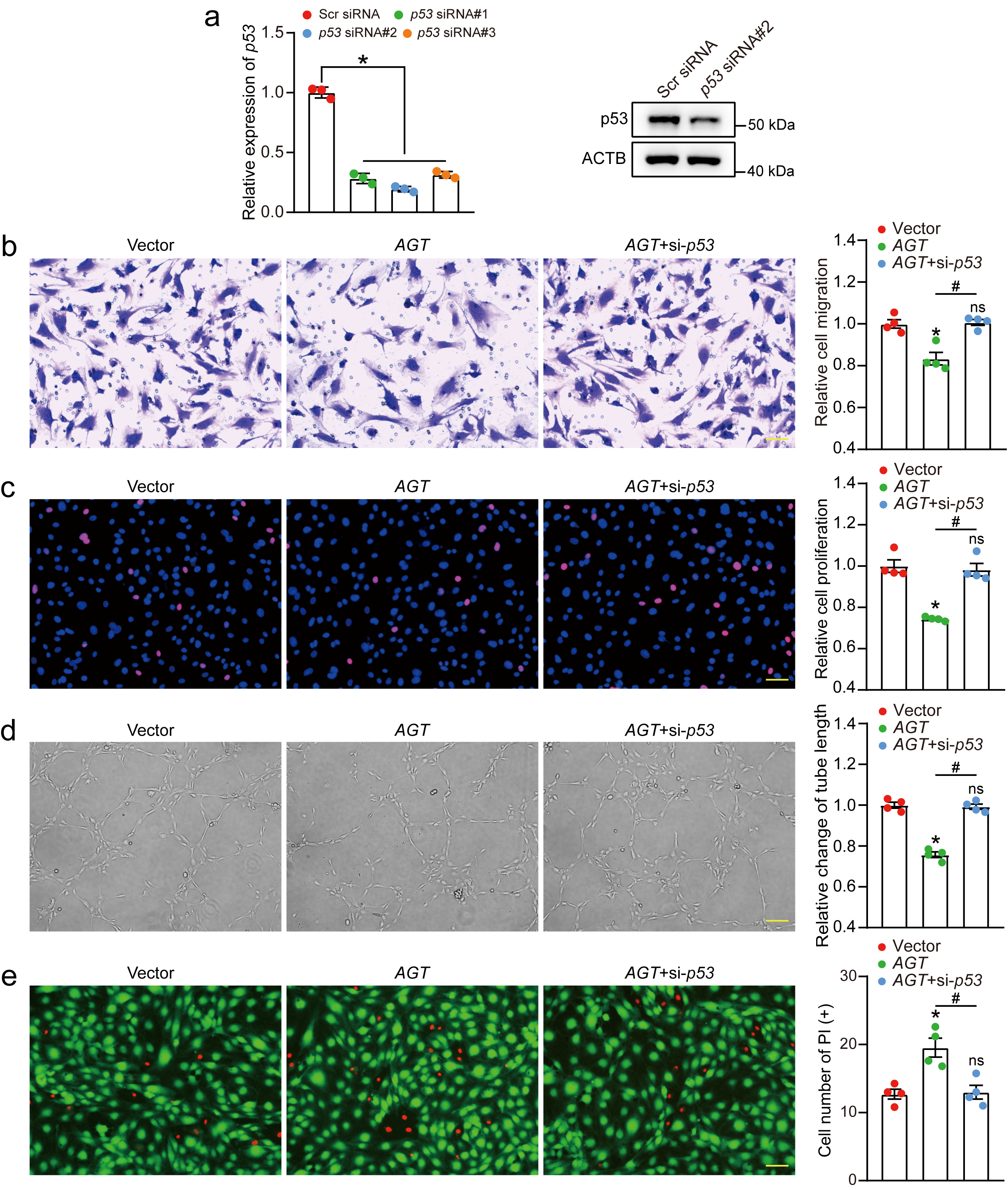
**

**Supplementary Figure 8. Knockdown of *p53* on the basis of *lnc-AGT-3* overexpression can restore cellular functions.**

*lnc-AGT-3* stable overexpression vector (*AGT*) or NC vector (Vector) were transfected into HUVECs, and then si-*p53* was transfected into *lnc-AGT-3* cells, generating *lnc-AGT-3* + si-*p53* cells. a. Verification of knockdown efficiency of *p53* siRNA. Left. The relative expression of *p53* was determined in HUVECs after *p53* siRNA (50 nM) transfection by qRT-PCR assays (n = 3, **P* < 0.05 vs. Scr siRNA, Student *t* test). Right. The relative expression of p53 was determined in HUVECs by western blotting (n = 4). b. Cell migration and quantitative analysis was conducted by transwell assays (n = 4, Scale bar, 50 μm). c. The proliferation ability of HUVECs was determined by EdU assays (n = 4, Scale bar, 50 μm). d. Tube formation assays were conducted to detect the tube formation ability of HUVECs (n = 4, Scale bar, 100 μm). e. After stimulating by H_2_O_2_ (300 μM) for 24 h, calcein-AM/PI assays were conducted to detect cell apoptosis (n = 4, Scale bar, 50 μm). **P* < 0.05 vs. Vector group; #*P* < 0.05 between the marked group; “ns” represents no statistical significance; One-way ANOVA followed by Bonferroni’s post hoc test.

**
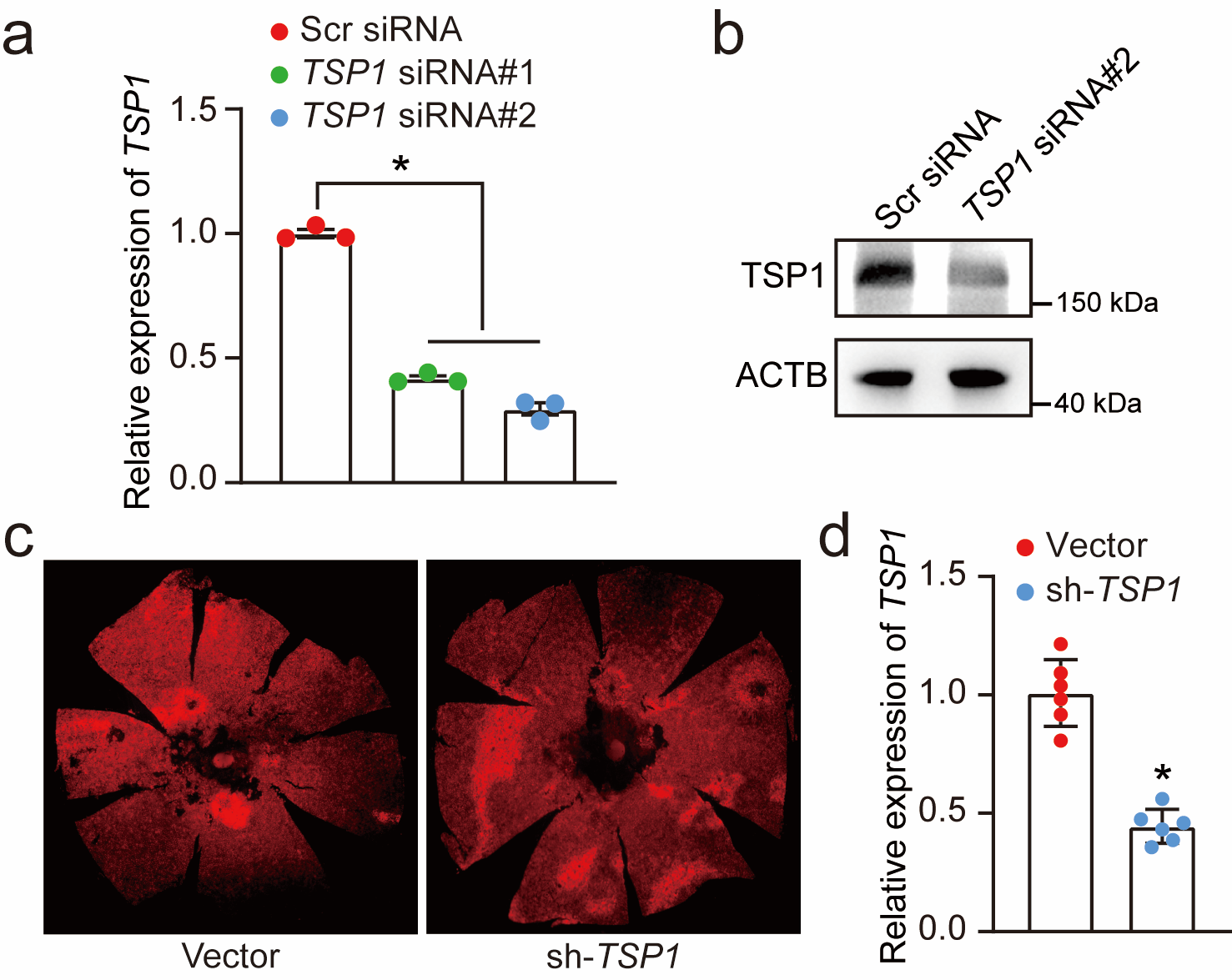
**

**Supplementary Figure 9. Verification of knockdown efficiency of *TSP1* *in vitro* and *in vivo*.**

a. The relative expression of *TSP1* was determined in HUVECs after *TSP1* siRNA (50 nM) transfection by qRT-PCR assays (n = 3, **P* < 0.05 vs. Scr siRNA, Student *t* test). b. The relative expression of TSP1 was determined in HUVECs by western blotting (n = 4). c. C57BL/6 mice received intravitreal injections of *TSP1* knockdownn scAAV (2 μL, 2.0E+9) for 5 days. The mice were euthanized, then the RPE/choroid complexes were dissected and flat-mounted for fluorescence observation (red). d. The levels of *TSP1* expression in choroid were detected by qRT-PCR assays (n = 6, **P* < 0.05 vs. Vector, Student *t* test).

**
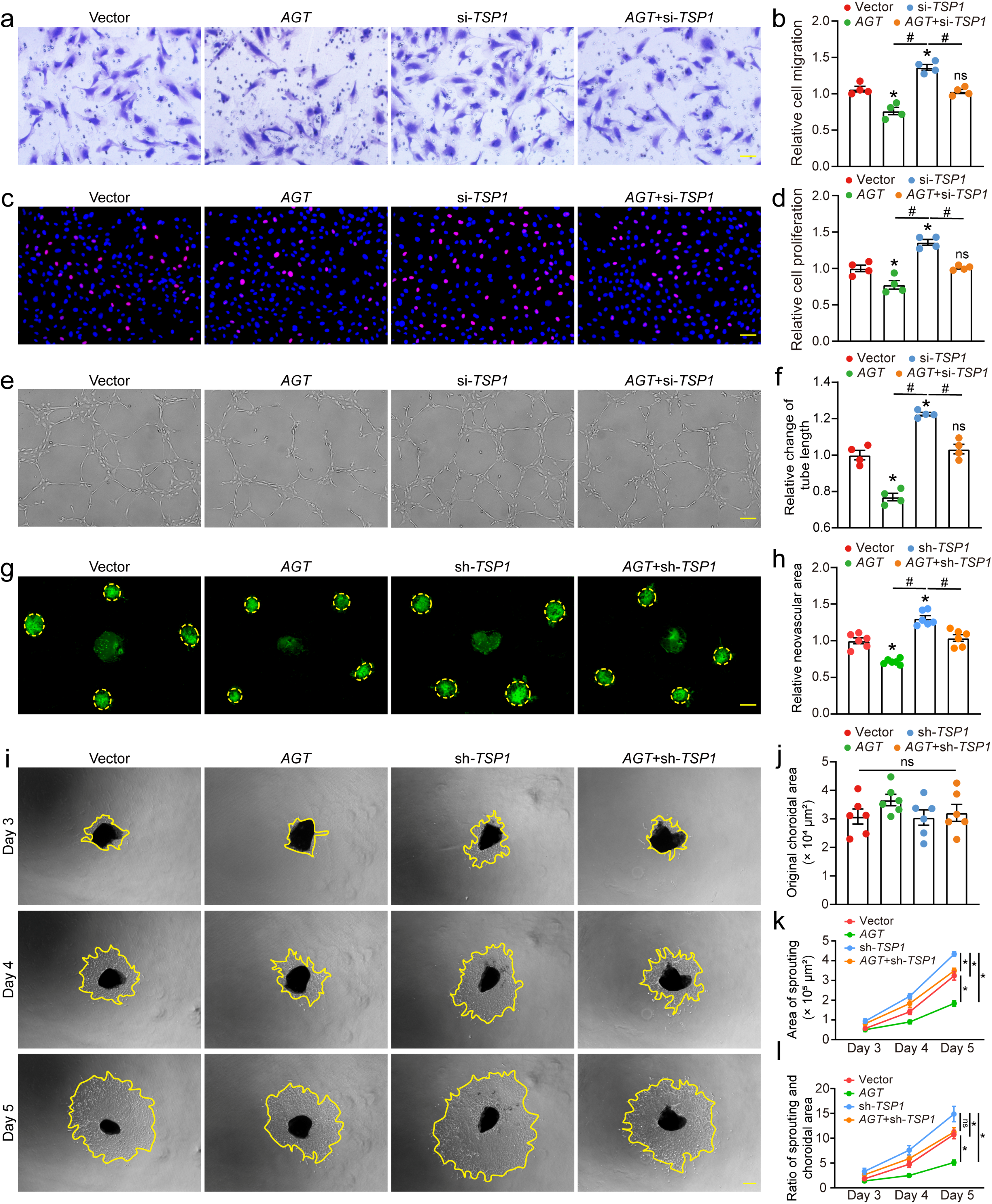
**

**Supplementary Figure 10. *TSP1* mediates *lnc-AGT-3*-driven anti-angiogenic effects *in vitro* and *in vivo*.**

*lnc-AGT-3* stable overexpression vector (*AGT*) or NC vector (Vector) were transfected into HUVECs or C57BL/6 mice, and then si-*TSP1* (50 nM) or sh-*TSP1* scAAV was added to generate *lnc-AGT-3* + si-*TSP1* cells or *lnc-AGT-3* + sh-*TSP1* mice. a-b. Cell migration and quantitative analysis was conducted by transwell assays (n = 4, Scale bar, 50 μm). c-d. The proliferation ability of HUVECs was determined by EdU assays (n = 4, Scale bar, 50 μm). e-f. Tube formation assays were conducted to detect the tube formation ability of HUVECs (n = 4, Scale bar, 100 μm). g-h. Laser photocoagulation were carried out at day 5 following the intravitreal injections. After 1 week, the mice were euthanized and the RPE/choroid complexes were dissected and flat-mounted for IB4 labeling. Yellow circles denote CNV lesions. Four spots per eye were averaged (n = 6, Scale bar, 200 μm). *P < 0.05 vs. Vector group; #P < 0.05 between the marked group; “ns” represents no statistical significance; ANOVA with Bonferroni. i-l. Choroidal sprouting assay: representative images at days 3-5 post-explant (Scale bar, 200 μm); quantification of sprouting potency (n = 5). *P < 0.05 vs. Vector group; ANOVA with Bonferroni.

**
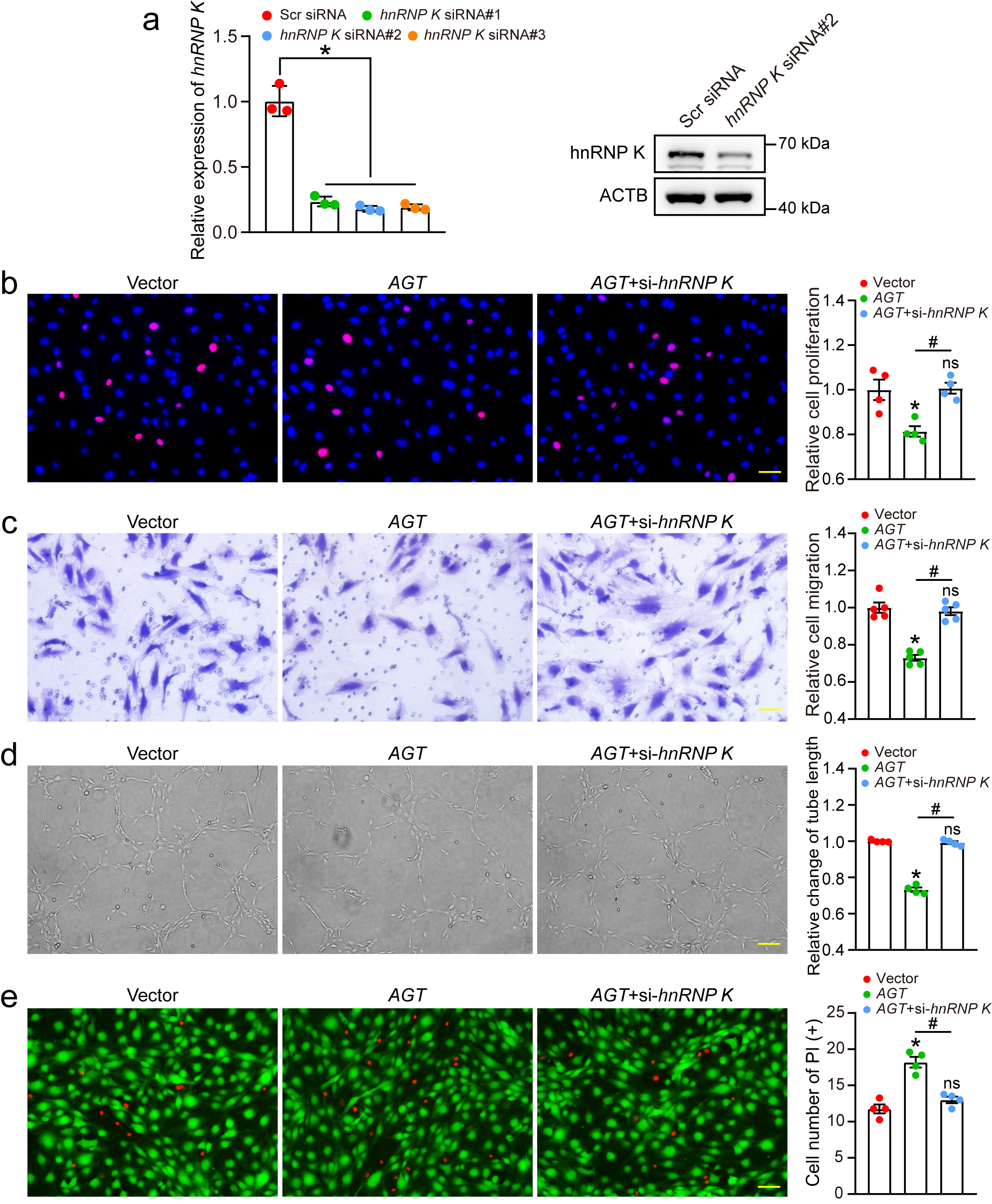
**

**Supplementary Figure 11. *hnRNP K* mediates *lnc-AGT-3*-driven HUVEC proliferation, migration, tube formation and apoptosis.**

*lnc-AGT-3* stable overexpression vector (*AGT*) or NC vector (Vector) were transfected into HUVECs, and then si-*hnRNP K* (50 nM) was transfected into *lnc-AGT-3* cells, generating *lnc-AGT-3* + si-*hnRNP K* cells. a. Verification of knockdown efficiency of *hnRNP K* siRNA. Left. The relative expression of *hnRNP K* was determined in HUVECs after *hnRNP K* siRNA (50 nM) transfection by qRT-PCR assays (n = 3, **P* < 0.05 vs. Scr siRNA, Student *t* test). Right. The relative expression of hnRNP K was determined in HUVECs by western blotting (n = 4). b. The proliferation ability of HUVECs was determined by EdU assays (n = 4, Scale bar, 50 μm). c. Cell migration and quantitative analysis was conducted by transwell assays (n = 5, Scale bar, 50 μm). d. Tube formation assays were conducted to detect the tube formation ability of HUVECs (n = 4, Scale bar, 100 μm). e. After stimulating by H_2_O_2_ (300 μM) for 24 h, calcein-AM/PI assays were conducted to detect cell apoptosis (n = 4, Scale bar, 50 μm). **P* < 0.05 vs. Vector group; #*P* < 0.05 between the marked group; “ns” represents no statistical significance; One-way ANOVA followed by Bonferroni’s post hoc test.

**Supplemental materials and methods**

**Immunofluorescent staining**

For retinal immunohistochemical analysis, enucleated eyes were first stabilized in Fekete's solution (4 °C, 2 h) before microdissection to remove anterior segment components. Posterior ocular cups were fixed in 4% PFA (Biosharp Biotechnology, China, BL539A) at 4 °C overnight, then cryoprotected in 30% sucrose and embedded in OCT medium for cryosectioning (5 μm thickness) using a Thermo Scientific cryostat. Sections mounted on adhesive slides (Citotest, China) underwent antigen retrieval and blocking (5% BSA/1% Triton X-100, 37 °C, 1h) prior to incubation with primary antibodies against NeuN (Abcam ab177487), rhodopsin (Abcam ab221664), and RBPMS (HUABIO, ER1901-43; all 1:200 dilution) at 4 °C overnight. After PBST washes, sections were labeled with species-matched fluorescent secondary antibodies (Invitrogen, A11012/A11005) for 2 h at RT, counterstained with DAPI, and imaged on an Olympus IX70 fluorescence microscope for quantitative analysis using ImageJ.

**Histology and Hematoxylin-Eosin staining (H&E staining)**

For comprehensive histological evaluation, enucleated eyes underwent fixation in FAS solution (Servicebio, G1109) for 24 hours followed by systematic tissue processing. Specimens were dehydrated through a graded ethanol series (75%-100%) and xylene clearing, then infiltrated with paraffin at 65 °C through three successive baths. Using a Leica RM2016 microtome, 4 μm sections were obtained and mounted on slides via a 40 °C flotation bath (KEDEE, KD-P), with subsequent drying at 60 °C. After standard H&E staining, slides were examined under an Olympus IX70 microscope for morphological assessment of retinal architecture.

**TUNEL assay**

Apoptotic cells in ocular sections were identified using a standardized TUNEL assay (Beyotime, China, C1086). Following deparaffinization and rehydration, tissue sections underwent antigen retrieval with proteinase K (20 μg/ml in 10mM Tris-HCl, RT, 15 min) before incubation with TUNEL reaction mix (37 °C, 1 h). Nuclei were counterstained with DAPI (5 min) to facilitate cellular localization, with fluorescent signal quantification performed on an Olympus microscope using image analysis software. This protocol enabled precise spatial mapping of apoptotic activity within distinct retinal layers.

**Cell apoptosis assay**

HUVEC apoptosis was systematically evaluated using complementary flow cytometric and fluorescence microscopy approaches. For quantitative assessment, cells (4×105/well in 6-well plates) were stained with Annexin V-FITC/PI (Vazyme, China, A211-01) in binding buffer (15min, RT, dark) and analyzed on a Beckman CytoFLEX flow cytometer (10,000 events/sample). Parallel samples were co-stained with PI/Calcein AM (Solarbio, P8080/AAT Bioquest, 22002) in 24-well plates (15-20 min, RT) for morphological evaluation of viable/apoptotic cells using fluorescence microscopy (Olympus IX73). This dual-method approach enabled both statistical quantification and visual confirmation of apoptotic progression.

**Spheroid sprouting assay**

A total of 125 ml of serum-free medium containing 3 g of methylcellulose (sigma, 9004-67-5) was mixed with 125 ml of 10% DMEM to prepare the methylcellulose solution. Suspended cells (at a density of 8 × 10⁴) were added to the dissolved methylcellulose solution, prepared by combining 1 ml of the methylcellulose solution with 4 ml of culture medium. The cell suspension was distributed in hanging drops on the underside of culture dish lids and inverted over PBS-containing wells to allow 24-hour spheroid formation through gravity-mediated cell aggregation. Individual spheroids were then embedded in rat collagen I matrix (R&D Systems, USA, 3440-100-01) and cultured for 24 h (37 °C, 5% CO_2_). Angiogenic sprouting was quantified by measuring the cumulative length of all cellular projections per spheroid using ImageJ software.

**Subcellular RNA fractionation and analysis**

To investigate compartment-specific gene expression, HUVECs were subjected to nuclear and cytoplasmic RNA isolation using the Norgen Biotek fractionation kit (NGB-21000). Following separation, RNA fractions were quantified and reverse-transcribed for qPCR analysis. Transcript localization was validated using ACTB mRNA (cytoplasmic marker) and U6 snRNA (nuclear control), enabling accurate normalization of subcellular gene expression patterns.

**CCK-8 assay**

Cell proliferation and viability were quantitatively assessed using the Cell Counting Kit‐8 (CCK8) (Beyotime, China, C0037). Cells were seeded in 96-well plates at 5×10³ cells/well (100 μL complete medium with 10% FBS) with six technical replicates per condition. Following treatment, 10 μL CCK-8 reagent was mixed with 90 μL serum-free DMEM to prepare the working solution, which was added to each well (100 μL/well). After 2 hours of incubation at 37 °C, metabolic activity was determined by measuring absorbance at 450 nm using a FilterMax F5 microplate reader (Molecular Devices), with values normalized to control wells.

**EdU assay**

Cellular proliferation kinetics were evaluated using the EdU detection kits (Beyotime, China, C0081S). Actively dividing cells were pulse-labeled with 10 μM EdU for 2 hours in 96-well culture plates, followed by fixation and fluorescent labeling according to the manufacturer's instructions. Quantitative analysis was performed using an Olympus IX73 DP80 fluorescence imaging system.

**Transwell migration assay**

HUVEC migratory capacity was evaluated using 8 μm pore transwell inserts (BD Falcon, 353097). A total of 1×10^5^ cells/100 μL (serum-free ECM) were placed in the upper chamber, with 500 μL complete medium (10% FBS) below. Following 24 h incubation (37 °C, 5% CO_2_), non-migrated cells were removed from the upper membrane surface. Transmigrated cells on the lower membrane were fixed with methanol, stained with 0.25% crystal violet (Macklin, China, C805211), and quantified in four random fields per insert using phase-contrast microscopy (Olympus IX73). Migration indices were calculated as mean cell counts normalized to control conditions.

**Tube formation assay**

The angiogenic capacity of HUVECs was evaluated using a Matrigel-based tube formation assay (BD Biosciences, USA, 356234). Growth factor-reduced Matrigel was polymerized in 24-well plates (37 °C, 20 min) before seeding 6×10^4^ treated cells/well in serum-free medium. Following 4-6 h incubation under standard culture conditions, capillary-like network formation was analyzed from five random microscopic fields (Olympus IX73) with ImageJ angiogenesis analyzer plugin.

**RNA isolation and quantitative real-time PCR (qRT-PCR) assay**

Total RNA extraction was performed using sample-specific purification kits (EZBioscience, USA): cellular RNA was isolated with the EZ-press RNA Purification Kit (B0004D), tissue-derived RNA with the Tissue RNA Purification Kit (RN001), and exosomal RNA with the Exosome RNA Purification Kit (exo-RN1). Concentration and purity of the RNA were evaluated using the NanoDrop ND-1000 Spectrophotometer (Nanodrop Technologies, USA). cDNA was synthesized and quantified using HiScript III All-in-one RT SuperMix Perfect for qPCR (Vazyme, China, R333-01). All synthesized cDNA samples were aliquoted and stored at -20 °C to preserve stability until analysis. Quantitative real-time PCR was performed using ChamQ SYBR Green Master Mix (Vazyme, Q321-02), with ACTB serving as the endogenous reference gene for normalization of target gene expression. Primer sequences for all analyzed transcripts are provided in Supplementary Table S6.

**Western blot**

Cellular and tissue proteins were extracted using RIPA lysis buffer supplemented with protease inhibitors (Selleckchem, USA, B14001), with protein concentrations determined by BCA assay. Equal amounts of protein (20 μg/lane) were resolved by SDS-PAGE and transferred to PVDF membranes. Following blocking with 5% non-fat milk (2 h, RT), membranes were probed with primary antibodies against p53 (10442-1-AP), hnRNP K (11426-1-AP), ubiquitin (10201-2-AP), TSP1 (67241-1-Ig; all from Proteintech) or ACTB (ab8227, Abcam) at 4 °C overnight, followed by HRP-conjugated secondary antibodies (Beyotime, A0208/A0216; 1h, RT). Protein signals were detected by ECL (SenBeiJia Biological Technology, China, BI-WB004) and quantified using ImageJ.

**Fluorecence in situ hybridization (FISH) and immunofluorescence (IF)**

RNA and protein subcellular distribution patterns were investigated through parallel FISH and IF assays. For FISH analysis, fixed cells (4% PFA) were permeabilized (0.5% Triton X-100, 4 °C) and hybridized with 20 μM RiboBio-synthesized probes (U6, 18S, lnc-AGT-3) overnight at 37 °C following 30 min pre-hybridization. Concurrent immunofluorescence staining involved incubation with anti-hnRNP K antibody (Proteintech, 11426-1-AP, 4 °C overnight) and FITC-conjugated secondary antibody (37 °C, 2 h) after standard fixation-permeabilization-blocking procedures. All samples were counterstained with DAPI and imaged under consistent parameters using an Olympus IX73 DP80 system, enabling simultaneous visualization of target transcripts and proteins.

**Immunoprecipitation (IP)**

For immunoprecipitation analysis, cells (5×10^6^ per 100 mm dish) were lysed and clarified by centrifugation (12,000 g, 10 min, 4 °C). Lysates were incubated with anti-p53 antibody (6 μL, Abcam, ab26) overnight at 4 °C, followed by Protein A/G magnetic bead capture (absin, Abs9649, 2 h, RT). Immunocomplexes were washed extensively and analyzed by western blotting to identify p53-interacting partners.

**Table S1: The information of ARC patients and nAMD patients involved in the study**

|  | ARC (n = 12) | nAMD (n = 12) | P value |
| --- | --- | --- | --- |
| Gender (Female/Male) | 6/6 | 6/6 | >0.9999 |
| Age, years (mean ± SEM) | 66.92±2.75 | 69.25±3.16 | 0.5830 |
| Hypertension, No (%) | 0 | 3 (25%) | 0.2174 |
| Diabetes, No (%) | 2 (16.67%) | 0 | 0.4783 |
| Dyslipidaemia, No (%) | 1 (8.33%) | 2 (16.67%) | >0.9999 |
| Smoking, No (%) | 3 (25%) | 5 (41.67%) | 0.6668 |

**Table S2: Target sequences of *lnc-AGT-3* smart silencer**

| Gene | Target sequences |
| --- | --- |
| *lnc-AGT-3* smart silencer | CCAACTACTTCAAAGGAAA |
|  | GACGAATGCAAGAATAGAA |
|  | CCATGAACCCAAACATAGA |
|  | AACACTGGGAAGGCAACAGT |
|  | GAAAGCAGTGAGACGAATGC |
|  | AACTCATGACCAACCAACTA |

**Table S3: All RNAi sequences used in this study**

| Gene | Sequence (5'-3') | Sequence (3'-5') |
| --- | --- | --- |
| Scramble siRNA | UUCUCCGAACGUGUCACGUTT | ACGUGACACGUUCGGAGAATT |
| *p53* siRNA1 | GAAGAGAAUCUCCGCAAGATT | UUCUUGCGGAGAUUCUCUUCTT |
| *p53* siRNA2 | GAGUAUUUGGAUGACAGAATT | UUCUGUCAUCCAAAUACUCTT |
| *p53* siRNA3 | CCAUCUACAAGCAGUCACATT | UGUGACUGCUUGUAGAUGGTT |
| *hnRNP K* siRNA1 | GGGUUGUAGAGUGCAUAAATT | UUUAUGCACUCUACAACCCTT |
| *hnRNP K* siRNA2 | CACUGAUGAGAUGGUUGAATT | UUCAACCAUCUCAUCAGUGTT |
| *hnRNP K* siRNA3 | GGUGAUCUUGGUGGACCUATT | UAGGUCCACCAAGAUCACCTT |
| *TSP1* siRNA1 | GGAGUUCAGUACAGAAAUATT | UAUUUCUGUACUGAACUCCTT |
| *TSP1* siRNA2 | CGAAUGUAGAGAUCCCUAATT | UUAGGGAUCUCUACAUUCGTT |

**Table S4: Primer sequences of *lnc-AGT-3* used in RNA-Pulldown**

| Gene |  | Sequence (3'-5') |
| --- | --- | --- |
| *lnc-AGT-3* Probe | *lnc-AGT-3*_NC | GTTTGACTGTCACACTCTGG |
|  | *lnc-AGT-3*_p1 | GTTCCCTGGTCCAGATAGTT |
|  | *lnc-AGT-3*_p2 | TATTCTTGCATTCGTCTCAC |

**Table S5: Top 5 proteins interacting with *lnc-AGT-3* identified by mass spectrometry**

| NO. | Entry name | MW (kDa) | Unique peptides | Score |
| --- | --- | --- | --- | --- |
| 1 | *HNRNPK* | 51 | 14 | 78.01 |
| 2 | *SFPQ* | 76 | 15 | 65.72 |
| 3 | *PTBP1* | 57 | 12 | 75.12 |
| 4 | *KRT6B* | 60 | 1 | 72.66 |
| 5 | *IGF2BP3* | 64 | 13 | 53.64 |

**Table S6: Primer sequences used for qPCR assays**

| Gene | Forward (5'-3') | Reverse (5'-3') |
| --- | --- | --- |
| *ACTB* | TTGTTACAGGAAGTCCCTTGCC | ATGCTATCACCTCCCCTGTGTG |
| *β-actin* | GGCTGTATTCCCCTCCATCG | CCAGTTGGTAACAATGCCATGT |
| *lnc-AGT-3* | ACCCAAACATAGACTTGCAAGACA | AGCACCAACATTGGCCTTTG |
| *lnc-RPL27A-5* | GCATCTACTTATGCAGAACCACA | ATATTGCTCCCACTGCAGTCTA |
| *lnc-CLDN16-7* | GCGGGTCAAGTGGCAGTAA | TGTGCACAACCCAGTTCAAGTC |
| *lnc-ST8SIA4-2* | AACCTGTTTCCAGGAAAGGAATG | CTTCTCTGGCAGACGTTCAG |
| *NONHSAG070941.2* | GACCCAGAAAAGTTTAGCACTGA | ACAGTGGAGCATCCCCTTC |
| *NONHSAG052946.2* | CCAGGAAACTAAGAGCAGGCA | CCCCTCCAGCAAGCTAAGAG |
| *lnc-ZFYVE1-8* | ACAGAAATGAGAACCACAGGC | GCCCACTCTTCTTGTTCATGT |
| *lnc-STIM2-10* | GGCACAATGAATCTGCATATGTTG | AGACACAGCCTAACTTCCATCA |
| *U6* | GCTTCGGGAGCACATATACTAAAAT | CGCTTCACGAATTTGCGTGTCAT |
| *Mus-lnc-agt-3* | GTCCTTCATCCTGGGAAGGC | ACCACCGAAGTGGGTTCTGA |
| *P53* | CAGCACATGACGGAGGTTGT | TCATCCAAATACTCCACACGC |
| *HNRNPK* | CAATGGTGAATTTGGTAAACGCC | GTAGTCTGTACGGAGAGCCTTA |
| *TSP1* | GCCATCCGCACTAACTACATT | TCCGTTGTGATAGCATAGGGG |
| *tsp1* | CCTGCCAGGGAAGCAACAA | ACAGTCTATGTAGAGTTGAGCCC |
